# Supplementary material for: Does a novel diagnostic pathway including blood-based risk prediction and MRI-targeted biopsies outperform prostate cancer screening using prostate-specific antigen and systematic prostate biopsies? - protocol of the randomised study STHLM3MRI
Source: BMJ Open. 2019 Jun 14;9(6):e027816. doi: 10.1136/bmjopen-2018-027816 (PMC6576112; doi:10.1136/bmjopen-2018-027816)
Supplement: Author's manuscript [file bmjopen-2018-027816.draft_revisions.pdf]

BMJ Open is committed to open peer review. As part of this commitment we make the peer review history of every article we publish publicly available.

When an article is published we post the peer reviewers' comments and the authors' responses online. We also post the versions of the paper that were used during peer review. These are the versions that the peer review comments apply to.

The versions of the paper that follow are the versions that were submitted during the peer review process. They are not the versions of record or the final published versions. They should not be cited or distributed as the published version of this manuscript.

BMJ Open is an open access journal and the full, final, typeset and author-corrected version of record of the manuscript is available on our site with no access controls, subscription charges or pay-per-view fees (http://bmjopen.bmj.com).

If you have any questions on BMJ Open's open peer review process please email info.bmjopen@bmj.com

BMJ Open

A study protocol for the randomized diagnostic study STHLM3MRI Main Study.

Journal:	*BMJ Open*
Manuscript ID	bmjopen-2018-027816
Article Type:	Protocol
Date Submitted by the Author:	20-Nov-2018
Complete List of Authors:	Nordstrom, Tobias; Karolinska Inst, Dpt Medical Epidemiology and Biostatistics<br>Jäderling, Fredrik; Karolinska Institutet, Molecular Medicine and Surgery<br>Carlsson, Stefan<br>Aly, Markus; Karolinska Institutet,<br>Grönberg, H; Karolinska Institutet,<br>Eklund, Martin; Karolinska Institutet,
Keywords:	Prostate disease < UROLOGY, Magnetic resonance imaging < RADIOTHERAPY, Urological tumours < UROLOGY

SCHOLARONE™
Manuscripts

Title:

A study protocol for the randomized diagnostic study STHLM3MRI Main Study.

Authors:

Tobias Nordström[a,b], MD PhD

Fredrik Jäderling[e,f], MD PhD

Stefan Carlsson[d,f], MD PhD

Markus Aly[a,d,f], MD PhD

Henrik Grönberg[a], MD PhD, Professor

Martin Eklund[a], PhD

[a]Department of Medical Epidemiology and Biostatistics, Karolinska Institutet, Sweden

[b]Department of Clinical Sciences at Danderyd Hospital, Karolinska Institutet, Sweden

[d]Department of Urology, Karolinska University Hospital Solna, Stockholm, Sweden

[e]Department of Diagnostic Radiology, Karolinska University Hospital, Stockholm, Sweden

[f]Department of Molecular Medicine and Surgery, Karolinska Institutet, Stockholm, Sweden

For peer review only

Correspondence to:

Tobias Nordström

Department of Medical Epidemiology and Biostatistics, Karolinska Institutet

S-171 77 Stockholm, Sweden

Email: tobias.nordstrom@ki.se

Phone: + 46 70 539 17 91

Keywords: Prostate cancer, Prostate neoplasm, biomarker, STHLM3, prostate biopsy, magnetic resonance imaging

1. *Abstract*

Introduction

Prostate cancer is a leading cause of cancer death among men in the Western world. Early detection of prostate cancer has been shown to decrease mortality, but has limitations with low specificity leading to unnecessary biopsies and over-diagnosis of low-risk cancers. The STHLM3 trial has paved the way for improved specificity in early detection of prostate cancer using the blood-based STHLM3 test for identifying men at increased risk of harbouring significant prostate cancer. Targeted prostate biopsies based on MRI images have been shown non-inferior sensitivity to detect significant prostate cancer and decrease the number of biopsies and non-significant cancers among men referred for prostate biopsy in clinical practice.

The overarching strategy of the STHLM3-MRI projects is to study an improved diagnostic pathway including an improved blood-based test for identification of men with increased risk of prostate cancer and use of MRI to select men for diagnostic workup with targeted prostate biopsies.

For peer review only - http://bmjopen.bmj.com/site/about/guidelines.xhtml

Methods

This is a study comparing traditional prostate cancer detection using PSA and systematic biopsies with the improved pathway for prostate cancer detection using the STHLM3 test and targeted biopsies in a screening context. The study will recruit 10,000 participants during 2018-2019 combining a paired and randomized design. This protocol follows SPIRIT guidelines. Endpoints include the number of detected prostate cancers, number of performed biopsy procedures and number of performed MRIs. Additional aims include to assess the health economic consequences and development of automated image-analysis.

Ethics and dissemination

The study has approval from the regional ethical board in Stockholm (2017-1280/31). Study findings will be published in peer-review journals. Findings will be also disseminated by conference/departmental presentations and by social and traditional media.

Registration details

ClinicalTrials.gov Identifier: NCT03377881

2. *Strenghts and limitations of this study*

- This is the first randomized study to examine the role of improved blood-based risk stratification used in sequence with MRI and targeted prostate biopsies in a screening-by-invitation context.
- The study examines the performance of the Stockholm3 test used together with MRI/Fusion technique compared with traditional PSA screening and will provide important data also on the performance of the Stockholm3 test or MRI/Fusion when used as standalone strategies.
- The study is performed at three study sites and uses centralized radiology and pathology.

For peer review only

3. Trial identifier

ClinicalTrials.gov Identifier: NCT03377881

4. Introduction

4.1. Public health significance of prostate cancer

Prostate cancer is the most common cancer and the leading cause of cancer death among men in Sweden. In year 2011 over 10,000 men were diagnosed with prostate cancer and more than 2,500 died due to the disease, approximately 20% of these in the Stockholm region. Prostate cancer incidence rates in Sweden are now comparable to rates in countries that had an early introduction of PSA testing, while prostate cancer mortality rates in Sweden are higher than in most other countries[1]. With over 90,000 prevalent cases, the health burden and the costs on the health care system are substantial. While a number of risk factors have been proposed for prevention of prostate cancer, including diet and occupational exposures, the only factors conclusively shown to increase risk of the disease are age, ethnicity and family history. Given the high prevalence of the cancer and limited opportunities for primary prevention, improved detection would reduce both procedure-related harm to men and economical cost in the healthcare system.

4.2. Early detection and treatment of prostate cancer: benefits and harms

The PSA test was first used to monitor disease progression in prostate cancer patients. The PSA test was taken up as a *de facto* screening test for prostate cancer in many countries, leading to rapid rises in prostate cancer incidence. The test characteristics for the PSA test in detecting prostate cancer are comparable to those for mammography for breast cancer screening, with a sensitivity of 72% and a specificity of 30-35% at a test threshold of 4 ng/ml[2]. However, a lower threshold of 3 ng/ml adopted in Sweden recently has led to increased sensitivity at the expense of reduced specificity. Recent analyses of PSA testing in the Stockholm area confirms these results showing that 46%, 68% and 77% of men 50-59, 60-69 and 70-79 years respectively have had at least one PSA test during a 9 years period[3].

Recent results from the large European Randomized Study of Screening for Prostate Cancer (ERSPC) including over 180,000 men provide increasing evidence that PSA screening has led to reduced mortality[4]. This report showed that PSA screening without digital rectal

For peer review only - http://bmjopen.bmj.com/site/about/guidelines.xhtml

examination was associated with a 21% relative reduction in the death rate from prostate cancer at a median follow-up of 11 years, with an absolute reduction of about 7 prostate cancer deaths per 10,000 men screened. Estimations from the ERSPC trial (men aged 55-69) show that 1,048 men would need to be offered screening and an additional 37 would need to be managed to prevent one prostate-cancer death during a 10-year period, leading to a significant overtreatment of indolent disease. The effectiveness of PSA testing was more marked at the Göteborg site of the ERSPC trial, with a risk reduction of 44% over 14 years in men aged 50-64[5]. This effect size is larger than that observed for mammographic screening for breast cancer and fecal occult blood testing for colorectal cancer.

However, using traditional systematic biopsies for diagnosis, approximately half of diagnosed cancers are low-risk tumors using the same main cutoff for biopsy as the ERSPC trial (PSA=3ng/ml) [6,7]. It has been shown that men with low-risk tumors treated without curative intent have the same survival as men in the background population[8], illustrating the large proportion of over-diagnosed cancers[9].

The STHLM3 study has shown a way to improve identification of men at increased risk of significant prostate cancer. Using the STHLM3 test, 32% of the prostate biopsies may be saved while not decreasing the sensitivity to high-grade disease (defined as Gleason Score ≥7) and simultaneously decreasing the number of low-grade tumors (Gleason Score ≤6) by 17%, thus decreasing overdiagnosis[7].

4.3. Traditional evaluation of men with increased risk of prostate cancer

Men at increased risk of prostate cancer - commonly estimated using PSA and palpatory findings - are traditionally assessed using systematic prostate biopsies. The procedure is performed under local anesthesia using antibiotic prophylaxis and includes 10-12 cores taken from predefined areas of the peripheral zone of the gland as visualized by endorectal ultrasound. While the biopsies systematically covers the prostatic gland rather than targeting a lesion, and non-lethal tumors are common, the risk of over-diagnosis (i.e. detection of non-significant tumors) is high [9]. The risk of non-representative biopsy findings result in underestimation of tumor grade compared with subsequent prostatectomy in up to 40% of men undergoing surgery[10]. The risk of severe post-biopsy infection has increased to 1-2% with increasing frequency of antibiotic resistance, further illustrating the need both to increase precision and decrease the number of performed biopsies[11].

For peer review only - http://bmjopen.bmj.com/site/about/guidelines.xhtml

4.4. Multi-parametric Magnetic Resonance Imaging (mpMRI) for detection of prostate cancer

Multi-parametric magnetic resonance imaging (mpMRI) incorporating anatomical and functional imaging has now been validated as a means of detecting and characterizing prostate tumors and can aid in risk stratification and treatment selection. The European Society of Urogenital Radiology (ESUR) in 2012 established the Prostate Imaging Reporting and Data System (PI-RADS) guidelines aimed at standardizing the acquisition, interpretation and reporting of prostate mpMRI. Consensus on an updated version (PI-RADS v2) have recently been published, outlining aspects of both interpretation and the technical execution[12-14]. Use of the revised PI-RADS provides moderately reproducible MR imaging scores for detection of clinically relevant disease[15]. Using MP-MRI to triage men might allow 27% of patients avoid a primary biopsy and diagnosis fewer clinically insignificant cancers. If subsequent TRUS-biopsies were directed by MP-MRI findings, up to 18% more cases of clinically significant cancer might be detected compared with the standard pathway of TRUS-biopsy for all[16].

In summary, PI-RADS recommends to use 3T or 1.5T machines, including T2- and T1-weighted sequences together with diffusion weighted images (DWI). Currently, the added value of dynamic contrast is not firmly established regarding tumor detection. At this time, there is no consensus among experts concerning the potential benefits of the use of endorectal coils for cancer detection. It has been suggested that the prevalence of suspicious lesions on MRI in men with clinical suspicion of prostate cancer is approximately 60% [17].

4.5. Targeted prostate biopsies guided by fusion technology

Targeted biopsies of the prostate consist of imaging (MRI) detecting significant tumors and a biopsy procedure where biopsies are targeted to the tumor using various devices for guidance[18]. While traditional endorectal ultrasound poorly identifies tumors, direction of biopsy needles can be performed in various ways. Cognitive or soft fusion is based on skilled urologists/radiologists interpreting the MRI images and directing needles solely based on the ultrasound images. The disadvantages of cognitive fusion lie in the potential for human error when attempting to mentally fuse the MRI with TRUS while aiming for cancers that are often <1 cm in diameter and the inability to track the location of each biopsy site. Hard fusion enables proper fusion of MRI information on the ultrasound image, possibly increasing precision.

For peer review only - http://bmjopen.bmj.com/site/about/guidelines.xhtml

Despite methodological flaws, a number of studies have investigated the value of fusion biopsies, primarily using non-randomized designs and non-screening populations[19]. In 2018, Kasi et al provided high quality evidence for men referred for prostate biopsy and showed that MRI/target biopsies are non-inferior for detection of significant cancer and decreases the number of in-significant cancers and number of biopsies as compared with systematic biopsies[20].

The proportion of men upgraded when comparing specimen from targeted biopsies and subsequent prostatectomy have been shown to be very low (<5%) whenusing targeted biopsies[21], increasing the proportion of men where treatment decisions are based on valid risk estimations.

4.6. Improving the diagnostic pathway for prostate cancer detection

The current diagnostic pathway for prostate cancer detection is characterized by several challenging hallmarks. First, testing with PSA is frequent also in men not benefitting from testing due to low PSA levels or high age[3]. Second, the currently used test for detection (PSA) lacks in specificity, resulting in frequent over-diagnosis[22,23]. Third, systematic biopsies shows high frequencies of benign tests, over-diagnosis, up-grading at prostatectomy, and risk of infectious complications[7,24]. Further, PSA testing increases with educational length and men with long education are more likely to have a prostate biopsy after an increased PSA value. These differences may contribute to the worse prostate cancer outcomes observed among men with lower socioeconomic status[25].

The STHLM3 test offers improved disease detection[7]. To further decrease over-detection, improve disease classification and spare men of test-related harm, prostate biopsy practice need to be improved. We hypothesize that an improved pathway for prostate cancer detection including a better blood-based screening test, improved selection to biopsy based on MRI findings and targeted biopsies guided by MRI/ultrasound fusion would dramatically decrease the number of biopsy procedures, overdiagnosis and improve treatment decisions.

5. Methods

For peer review only - http://bmjopen.bmj.com/site/about/guidelines.xhtml

5.1. Hypotheses

5.1.1. Primary hypotheses

The below hypothesis is posed for men in screening-by-invitation context:

A diagnostic pathway using the Stockholm3 test to select men for further workup using MRI followed by targeted biopsies and systematic biopsies (S3M-MR-TBx/SBx) has non-inferior sensitivity for detecting clinically significant cancer (ISUP grade group ≥ 2) and reduces the number of performed biopsy procedures compared to a diagnostic pathway using systematic biopsies in men with PSA ≥3 ng/ml (PSA-SBx).

5.1.2. Additional hypotheses

1. As compared with performing systematic biopsies for men with elevated risk of prostate cancer in prostate cancer screening, targeted prostate biopsies performed with MRI/Fusion technique with or without addition of systematic biopsies has non-inferior sensitivity for detecting clinically significant cancer (ISUP grade group ≥ 2) and reduces the number of performed biopsy procedures.
2. A diagnostic pathway using the Stockholm3 test to select men for further workup using MRI followed by ONLY targeted biopsies (S3M-MR-TBx) has non-inferior sensitivity for detecting clinically significant cancer (ISUP grade group ≥ 2) and reduces the number of performed biopsy procedures compared to a diagnostic pathway using systematic biopsies in men with PSA ≥3 ng/ml (PSA-SBx).
3. Adding prostate volume as parameter in the diagnostic pathway with Stockholm3 test and MRI/Fusion biopsies improves model precision.
4. A diagnostic pathway with Stockholm3 followed by MRI and targeted biopsies has non-inferior sensitivity for detecting clinically significant cancer (ISUP grade group ≥ 2) and reduces the number of MRI examinations and performed biopsies compared to a diagnostic pathway using PSA ≥3 ng/ml followed by MRI and targeted biopsies.
5. SBx in the MRI arm has superior sensitive than SBx in the non-MRI arm (due to cognitive fusion).
6. Biopsy compliance is higher after biopsy is recommended based on MRI compared to recommended without MRI.

For peer review only - http://bmjopen.bmj.com/site/about/guidelines.xhtml

7. A diagnostic pathway using the Stockholm3 test to select men for further workup using MRI and targeted biopsies (S3M+TBx) shows better health economy (positive ICER) compared to a diagnostic pathway using systematic biopsies in men with PSA ≥3 ng/ml (PSA+SBx).

5.2. Aims

To compare a diagnostic pathway using the Stockholm3 test (S3M ≥ 11%) to select men for further workup using MRI (PI-RADS ≥ 3) and targeted biopsies (S3M+TBx) to a diagnostic pathway using systematic biopsies in men with PSA ≥3 ng/ml (PSA+SBx) with respect to number of diagnosed clinically significant cancer (ISUP grade group ≥ 2) and number of performed biopsies. Additional aims corresponding to hypotheses 2-8 above will be assessed.

5.3. Study design

STHLM3-MR Phase 2 is a study combining a paired and a randomized design (Figure 1). The study will follow the following outline: Participants will be invited by mail. All participants will undergo a blood-test, including PSA and the STHLM3 test. Men with an elevated PSA ≥3 ng/ml *or* PSA ≥1.5ng/ml and S3M>11% will be randomized to either traditional prostate biopsies or MR with targeted biopsies on MR lesions.

5.4. Participants, interventions and outcomes

5.4.1. Study setting

This is a screening-by-invitation study including one study administrative center, two radiological sites and three urological sites where data will be collected.

Participating urological centras

Department of Urology, Capio St Görans Hospital: dr Henrik Grönberg
Uroclinic, Sophiahemmet, Stockholm; dr Olof Jansson
Odenplans läkarhus; dr Magnus Annerstedt

For peer review only

5.4.2. Eligibility criteria

Inclusion criterias

Men age 50-74 years without prior diagnosis of prostate cancer (ICD-9 C61).

Permanent postal address in Stockholm

Not a previous participant in the Stockholm3 study (2012-2014)

Exclusion criterias

Severe illnesses such as metastatic cancers, severe cardio-vascular disease or dementia

Contraindications for magnetic resonance imaging (MRI) eg pacemaker, magnetic cerebral clips, cochlear implants or severe claustrophobia.

Men with a previous prostate biopsy the preceding 60 days before invitation.

5.4.3. Randomization

Randomization is performed 2:3 between control arm and experimental arm. Randomization will be performed will be performed using stratification on disease risk [6 stratas]. Disease risk is assessed using the Stockholm3 test. Test are discordant if PSA is negative and Stockholm3 positive or vice versa.

Four allocation lists [high/low risk vs discordant/concordant tests] have been created with the sequence [control arm, control arm, experimental arm, experimental arm, experimental arm]. Participants are first allocated to corresponding list, and then allocated to study arm according to the order in which they participate. The allocation sequence is blinded for the study investigators and handled by the study database administrator (A Björklund).

In order to enhance resource usage, men are allocated to the study sites according to local availiability of biopsy procedure slots.

5.4.4. Interventions

Blood sampling

For peer review only - http://bmjopen.bmj.com/site/about/guidelines.xhtml

Participating men undergo blood-sampling with analysis of PSA and the Stockholm3 test at Karolinska University Laboratory.

For the main analysis, the Stockholm3 test include clinical data as answered when consenting participation (previous biopsy, age, finasteride medication, relatives with prostate cancer); single nucleotide polymorphisms and measurements of protein levels (MSMB, MIC1, PSA, fPSA, hK2). For secondary analyses, clinical information on DRE and prostate volume is included. The algorithm for calculation of the Stockholm3 test result has been described (Ström et al, European Urology 2018).

Definition of EXPERIMENTAL ARM

Men randomized to the experimental arm undergoes MRI. If suspicious lesions are found, the participant undergoes targeted biopsies using Fusion technology *followed by systematic biopsies.*

Men without lesions are excepted from further intervention and receives notification on recommendation for follow-up. Technology and process are described below.

Men with a Stockholm3 risk ≥25% and no suspicious lesion on MRI will undergo systematic biopsies.

Definition of CONTROL ARM

Men randomized to the control arm undergoes systematic biopsies as defined below.

Technology

Cut-offs for performing the STHLM3 test

The STHLM3 test will be performed for men with a PSA ≥ 1.5 ng/ml

Cut-offs for entering randomization

Participants with PSA ≥ 3.0 ng/ml or STHLM3-test ≥ 11% risk of Gleason Score ≥7 cancer will be randomized and offered to undergo either MR or systematic biopsies (See Process description).

MRI technology

Location and MRI equipment

Capio St Görans Hospital: General Electric, Architect, 3T

Globen Unilabs Healthcare: Siemens Magnetom Aera 1.5T

For peer review only - http://bmjopen.bmj.com/site/about/guidelines.xhtml

Patient preparations

Refraining from sexual activity with ejaculation 3 days prior to examination

Fasting patient 6 h

Minimal preparation enema prior to examination

Antispasmodic agent (Glucagon) just before the examination

MRI Protocol

A short (14 minutes) MRI protocol will be used. A detailed description is available. Briefly, the protocol includes: T2w images axial, sagittal, coronal; Diffusion weighted imaging b0 and b1000 with ADC and a synthetic b1500 limited to the prostate location; No endorectal coil will be used.

MRI Interpretation

MRI interpretation is centralized to Capio St Görans hospital and is performed according to PIRAD v2.0 for examinations without adequate perfusion studies. Dr Fredrik Jäderling is responsible for MRI interpretation. Dr Jäderling or 1-2 other, experienced radiologists at his department performs all MRI interpretations.

PI-RADS v2 ("Assessment without adequate dynamic contrast enhanced imaging") will be used, with a 1-5 grade scale of suspicious lesions (1= clinically significant cancer is highly unlikely to be present, 5= clinically significant cancer is highly likely to be present).

During the study period participating radiologist will have access to updated histology results of fusion biopsies to be able to adjust their MRI reading according to tumor detection rates for different PIRAD diagnoses as defined above.

Fusion biopsy technology

Brand/models

BK Medical (BK Ultrasound ; www.bkultrasound.com/bk-medical/fusion)

The BK Medical fusion system is the only fusion device compatible with BK Medicals ultrasound devices, used by the urology departments participating in the study. The system represents a second generation ultrasound system with integrated MRI Fusion. MRI data is imported through HIPAA-compliant PACS connection with the local radiology department.

Definition of targeted biopsies

Using MRI data with pre-marked borders of the prostate and tumor, fusion of MRI images and ultrasound images are performed bedside. Using local anesthetic and antibiotic

For peer review only - http://bmjopen.bmj.com/site/about/guidelines.xhtml

prophylaxis, lesions are according to below. Targeted biopsies are always combined with systematic biopsies.

Biopsy procedure for targeted biopsies

PI-RADS≥3: 3-4 targeted biopsies on marked lesions + systematic biopsies

Large diffuse lesions or poor image quality: Systematic biopsies including lesion

No PI-RADS≥3, diffuse lesions and at least acceptable image quality: No biopsies are performed.

In larger lesions in PI-RADS category 3 and 5, areas within the lesion with the lowest ADC value ("Target-within-target") will be targeted with the first biopsy taken from the lesion, to evaluate the additional value regarding tumor staging.

Definition of systematic biopsies

10-12 systematic biopies are taken from the peripheral zone as previously described in STLHLM3 and the National Guidelines. Extra biopsies are allowed from additional sites visible on ultrasound or according to palpatory findings. In summary, systematic biopsies are performed in the peripheral zone as 4 lateral and para-median biopsies on the left and right side, in the base and mid part of the gland. In the apical third of the gland one lateral left and right biopsy is performed.

Pathology

Pathology is centralized to Unilabs/Capio St Görans hospital. Dr Axel Glaessgen is responsible for the integrity of analyzes of pathological specimen. 2-3 uro-pathologists at dr Glaessgens department assesses all pathological specimen with intermittent cross-validation between them. Pathology preparation and reporting follow ISUP 2014 guidelines.

The pathology preparation is done by Unilabs as part of the normal clinical routine. Biopsy specimens are analyzed according to local practice.

Localisation of biopsies in the prostate are described using Swedish National Guideline nomenclature (A1-4; B1-4; C1-4; anterior/posterior). Gleason Score, mm cancer and % Gleason 4 is reported on each needle specimen.

Pathologist notes results in the usual way in the laboratory system. The result of the pathological analysis is submitted in accordance to existing clinical routines to the referring urologist. A copy of the result is delivered to the study administration.

5.4.5. Outcomes

For peer review only - http://bmjopen.bmj.com/site/about/guidelines.xhtml

There are three co-primary endpoints in this trial:

Number of diagnosed ISUP grade group ≥ 2 cancers

Number of diagnosed ISUP grade group 1 cancers

Number of performed biopsies

5.4.6. Follow-up

Main study outcomes are assessed after prostate biopsy procedures. Additional participant data will be secured in the following circumstances:

No suspicious lesion on MRI:

Men in the experimental arm without suspicious lesions on MRI will be informed and recommended follow-up by the responsible, local urologist. After additional ethical application, the co-investogators might initiate retrospective follow-up of these participants.

Men with diagnosed prostate cancer

Participants with prostate cancer diagnosed on biopsy within the study will be followed up after the biopsy to secure data on the following: Treatment modality (Active Surveillance, Surgery, Radiation); Treatment lead-time and site; Pathological report after surgery (positive margins, T-stage, etc). Data will be assessed through medical records intermittently.

5.5. Serious adverse events

Study nurse will monitor serious adverse events after the prostate biopsy procedures. To ensure this, the study nurse will follow this check medical journals for hospitalization within 1 week after the biopsy procedure in the journal systems Take Care and Cosmic (covering the main part of hospitals in Stockholm region). This will be initiated as individual biopsy results are registered at the study administration. Results will be provided to the Data Safety and Monitoring Board.

5.6. Participant timeline

Figure 2 illustrates the approximate timeline for participating men in STHLM3MRI Main Study.

For peer review only

5.7. Sample size

STHLM3-MR/Fusion Phase 2 will invite 25,000 men and aim to include 10,000 participants (see **Error! Reference source not found.**). We anticipate to perform 1,039 biopsy procedures altogether. Inclusion will continue until complete data on 415 men in the control arm (SBx) and 623 men in the experimental arm (MR-TBx-SBx).

Basic data and assumptions used in the sample size calculations

We used data from the STHLM3 trial [REF Grönberg et al. Lancet Oncology 2015] for sample size calculations. In this data, 18% of men with PSA ≥ 3 had a clinically significant prostate cancer when biopsied with SBx. We further noted that rTPR=1.45 for clinically significant prostate cancer comparing MRI+TBx with SBx based on the results from the PRECISION randomized trial [REF Kasivisvanathan et al. NEJM 2018]. However, we will for sample size calculations use rTPR=1.25 for MRI+TBx vs. SBx as a more conservative estimate. We set the nonferiority delta to 4 percentage points for demonstrating noninferiority with respect to sensitivity of clinically significant prostate cancer. We set the alpha to 5%.

Primary contrast

Simulating 1000 trials (by bootstrapping from the STHLM3 data) under the assumptions outlined in the preceding section 303 men need to biopsied in the SBx arm based on PSA ≥ 3 to have 80% power to demonstrate non-inferior sensitivity of S3M+MRI+TBx compared with PSA+SBx. This means that at least **415** men need to be biopsied in the SBx arm (since some men are not randomized based on PSA ≥ 3 but on S3M ≥ 11%) and, consequently, **623** to the MRI arm (because of the 2:3 randomization). Total number of men undergoing workup according to protocol (SBx in the no MRI arm and MRI and TBx if Pi-RADS ≥ 3 in the MRI arm) is thus 1038. Assuming 20% dropout, 1300 men need to be randomized. These numbers give 80% power to detect a modest 17% reduction in biopsies between the two strategies.

5.8. Recruitment and Process Description

The STHLM3-MR Phase 2 will use existing solutions developed and optimized in the previous studies STHLM3 and STHLM3-MR Phase 1 where all major components of the process have been tested. First, participants will follow the *paired design study process* where inclusion, blood-test and delivery of recommendation letter is performed. Men with increased risk of high-grade prostate cancer then enter the *randomized study process*, where extended work-up including biopsies are performed.

For peer review only - http://bmjopen.bmj.com/site/about/guidelines.xhtml

5.9. Data Collection, management, analysis

5.9.1. Data collection

Primary data sources are

i. clinical variables collected from laboratory referral
ii. biopsy referrals and reports
iii. pathology reports
iv. MRI reports
v. blood concentrations of kallikreins, MSMB, MIC1, SNPs

Collection of i. – iv. is performed by study nurses (C Cavalli-Björkman) on a weekly basis from participating urology sites, participating radiologists. For v., this is digitally transferred from Karolinska University Laboratory.

5.9.2. Data management

Data is collected, entered, coded and stored at Department of Medical Epidemiology and Biostatistics, Karolinska Institutet. Data is entered by Study Nurse using predefined database sheets developed in STHLM3MRI Phase 1. This is blinded from study co-investigators and data is stored at the department under supervision by the study database administrator (SDA, Astrid Björklund). Any extraction of study data is performed by the SDA after approval of PI Tobias Nordström.

5.9.3. Data analysis

Analysis of data is described in the Statistical Analysis Plan (SAP).

5.9.4. Auditing and Monitoring

A Data Safety and Monitoring Board (DSMB) is assembled and consist of dr Hans Garmo (Statistician), prof Ola Bratt (Urology) and prof Holmberg (Urology/Study Design). The DSMB audits protocol and process descriptions and one interim data extraction performed by the study database administrator after 10% (100 men) have completed the control or experimental arms. The co-investigators are blinded to the interim data and analysis results. The work of the DSMB is regulated in the DSMB Charter.

5.10. Patient and Public Involvement

For peer review only - http://bmjopen.bmj.com/site/about/guidelines.xhtml

The research question and outcome measures were designed to improve prostate cancer diagnostics. This includes optimizing prostate biopsies and decreasing over-detection, both associated with morbidity. Patient organisations were informed on the results from the STHLM3MRI Phase 1 study. Patients were not involved in recruitment of the study. Results will be disseminated to participants through common and scientific channels.

6. Ethics and dissemination

6.1. Research ethics approval

The study has approval from the regional ethical board in Stockholm (2017-1280/31).

6.2. Consent

Participant consent is secured when the participant is included to the study at www.kliniskastudier.se. This includes secure identification using Mobilt BankID. Additional approval on use of biological specimen data is collected on the biopsy referral.

6.3. Confidentiality

Study data is collected and stored at Department of Medical Epidemiology and Biostatistics, Karolinska Insitutet using secure Oracle servers. All data extractions are made by database administrator and are anonymized (personal id number is removed) before dissemination to researchers.

6.4. Dissemination

Analyses results on the posed aims will be submitted for peer-reviewed publication and submitted for presentation at scientific congress. Communication of the results will be made to patient organisations (Prostatacancerförbundet) and non-scientific channels. No use of professional writers are planned.

The study protocol is made publicly availiable through clinicaltrials.gov.

6.5. Data Sharing Statement

For peer review only

Anomymized, individual participatant data that underlie the results reported in this article, after deidentification (text, tables, figures and appendices) will be available for data sharing. Proposals may be submitted up to 36 months following article publication. Data will be shared with investigators whose proposed use of the data has been approved by an independent review committee identified for this purpose.

7. Declarations of interest

Henrik Grönberg has five prostate cancer diagnostic related patents pending, has patent applications licensed to Thermo Fisher Scientific, and might receive royalties from sales related to these patents. Martin Eklund is named on four of these five patent applications. Karolinska Institutet collaborates with Thermo Fisher Scientific in developing the technology for the Stockholm3 test.

8. Contributions

TN was the Principal Investigator. TN, HG, ME, SC and MA designed the study. ME and TN interpreted preliminary data. FJ designed MRI protocols and collected data.

We thank participants, study organizers, participating researchers and clinicians, and patient advisers for their contributions to the STHLM3MRI project.

9. Funding statement

Funding was provided by the Swedish Cancer Society, (Cancerfonden), the Swedish Research Council (Vetenskapsrådet), Swedish Research Council for Health Working Life and Welfare (FORTE), The Strategic Research Programme on Cancer (StratCan), Karolinska Institutet, Swedish e-Science Research Center (SeRC) and Stockholm City Council (SLL). The STHLM3 study is a part of the Linnaeus Center CRISP ‘‘Predication and prevention of breast and prostate cancer’’ funded by the Swedish Research Council.

10. Figure legends

Figure 1: Study design overview STHLM3MRI Main Study

For peer review only - http://bmjopen.bmj.com/site/about/guidelines.xhtml

Figure 2: Timeline overview for study participants in STHLM3MRI Main Study

11. References

1 Ferlay J, Soerjomataram I, Dikshit R, *et al.* Cancer incidence and mortality worldwide: Sources, methods and major patterns in GLOBOCAN 2012. *Int J Cancer* 2014;:n/a–n/a. doi:10.1002/ijc.29210

2 Mistry K, Cable G. Meta-analysis of prostate-specific antigen and digital rectal examination as screening tests for prostate carcinoma. *J Am Board Fam Pract* 2003;**16**:95–101.

3 Nordström T, Aly M, Clements MS, *et al.* Prostate-specific antigen (PSA) testing is prevalent and increasing in Stockholm County, Sweden, Despite no recommendations for PSA screening: results from a population-based study, 2003-2011. *Eur Urol* 2013;**63**:419–25. doi:10.1016/j.eururo.2012.10.001

4 Schröder FH, Hugosson J, Roobol MJ, *et al.* Prostate-cancer mortality at 11 years of follow-up. *N Engl J Med* 2012;**366**:981–90. doi:10.1056/NEJMoa1113135

5 Hugosson J, Carlsson S, Aus G, *et al.* Mortality results from the Göteborg randomised population-based prostate-cancer screening trial. *Lancet Oncol* 2010;**11**:725–32. doi:10.1016/S1470-2045(10)70146-7

6 Nationell kvalitetsrapport för diagnosår 2012. Regionala Cancercentrum i Samverkan 2013. http://npcr.se/wp-content/uploads/2013/04/20131121-NPCR-Rapport-2012.pdf

7 Grönberg H, Adolfsson J, Aly M, *et al.* Prostate cancer screening in men aged 50-69 years (STHLM3): a prospective population-based diagnostic study. *Lancet Oncol* 2015;**16**:1667–76.

8 Rider JR, Sandin F, Andrén O, *et al.* Long-term outcomes among noncuratively treated men according to prostate cancer risk category in a nationwide, population-based study. *Eur Urol* 2013;**63**:88–96. doi:10.1016/j.eururo.2012.08.001

9 Loeb S, Bjurlin MA, Nicholson J, *et al.* Overdiagnosis and overtreatment of prostate cancer. *Eur Urol* 2014;**65**:1046–55. doi:10.1016/j.eururo.2013.12.062

10 Soares R, Di Benedetto A, Dovey Z, *et al.* Minimum 5-year follow-up of 1138 consecutive laparoscopic radical prostatectomies. *BJU Int* 2015;**115**:546–53. doi:10.1111/bju.12887

11 Aly M, Dyrdak R, Nordström T, *et al.* Rapid increase in multidrug-resistant enteric bacilli blood stream infection after prostate biopsy-A 10-year population-based cohort study. *Prostate* 2015;**75**:947–56. doi:10.1002/pros.22979

For peer review only

12 Barrett T, Turkbey B, Choyke PL. PI-RADS version 2: what you need to know. *Clin Radiol* 2015;**70**:1165–76. doi:10.1016/j.crad.2015.06.093

13 Barentsz JO, Weinreb JC, Verma S, *et al.* Synopsis of the PI-RADS v2 Guidelines for Multiparametric Prostate Magnetic Resonance Imaging and Recommendations for Use. *Eur Urol* 2015;**69**:41–9. doi:10.1016/j.eururo.2015.08.038

14 Weinreb JC, Barentsz JO, Choyke PL, *et al.* PI-RADS Prostate Imaging - Reporting and Data System: 2015, Version 2. *Eur Urol* 2015;**69**:16–40. doi:10.1016/j.eururo.2015.08.052

15 Muller BG, Shih JH, Sankineni S, *et al.* Prostate Cancer: Interobserver Agreement and Accuracy with the Revised Prostate Imaging Reporting and Data System at Multiparametric MR Imaging. *Radiology* 2015;**277**:741–50. doi:10.1148/radiol.2015142818

16 Ahmed HU, El-Shater Bosaily A, Brown LC, *et al.* Diagnostic accuracy of multi-parametric MRI and TRUS biopsy in prostate cancer (PROMIS): a paired validating confirmatory study. *Lancet* Published Online First: 19 January 2017. doi:10.1016/S0140-6736(16)32401-1

17 Moore CM, Robertson NL, Arsanious N, *et al.* Image-guided prostate biopsy using magnetic resonance imaging-derived targets: a systematic review. *Eur Urol* 2013;**63**:125–40. doi:10.1016/j.eururo.2012.06.004

18 Sonn GA, Margolis DJ, Marks LS. Target detection: magnetic resonance imaging-ultrasound fusion-guided prostate biopsy. *Urol Oncol* 2014;**32**:903–11. doi:10.1016/j.urolonc.2013.08.006

19 Schoots IG, Roobol MJ, Nieboer D, *et al.* Magnetic Resonance Imaging-targeted Biopsy May Enhance the Diagnostic Accuracy of Significant Prostate Cancer Detection Compared to Standard Transrectal Ultrasound-guided Biopsy: A Systematic Review and Meta-analysis. *Eur Urol* 2014;**68**:438–50. doi:10.1016/j.eururo.2014.11.037

20 Kasivisvanathan V, Rannikko AS, Borghi M, *et al.* MRI-Targeted or Standard Biopsy for Prostate-Cancer Diagnosis. *N Engl J Med* 2018;:NEJMoa1801993. doi:10.1056/NEJMoa1801993

21 Baco E, Ukimura O, Rud E, *et al.* Magnetic resonance imaging-transectal ultrasound image-fusion biopsies accurately characterize the index tumor: correlation with step-sectioned radical prostatectomy specimens in 135 patients. *Eur Urol* 2015;**67**:787–94. doi:10.1016/j.eururo.2014.08.077

22 Arnsrud Godtman R, Holmberg E, Lilja H, *et al.* Opportunistic Testing Versus Organized Prostate-specific Antigen Screening: Outcome After 18 Years in the Göteborg Randomized Population-based Prostate Cancer Screening Trial. *Eur Urol* 2014;**68**:354–60. doi:10.1016/j.eururo.2014.12.006

For peer review only - http://bmjopen.bmj.com/site/about/guidelines.xhtml

23 Thompson IM, Pauler DK, Goodman PJ, *et al.* Prevalence of prostate cancer among men with a prostate-specific antigen level. *N Engl J Med* 2004;**350**:2239–46. doi:10.1056/NEJMoa031918

24 Loeb S, Vellekoop A, Ahmed HU, *et al.* Systematic review of complications of prostate biopsy. *Eur Urol* 2013;**64**:876–92. doi:10.1016/j.eururo.2013.05.049

25 Nordström T, Bratt O, Örtegren J, *et al.* A population-based study on the association between educational length, prostate-specific antigen testing and use of prostate biopsies. *Scand J Urol* 2016;**50**:104–9. doi:10.3109/21681805.2015.1113200

For peer review only

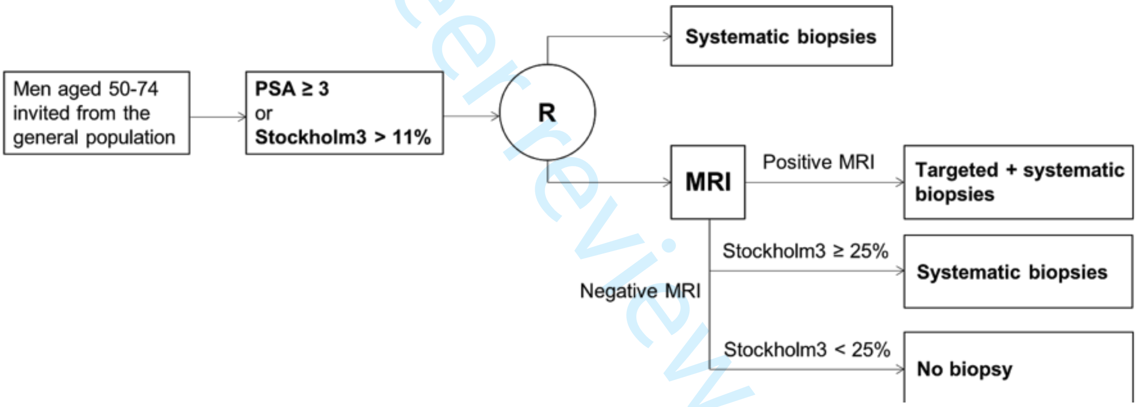

For peer review only

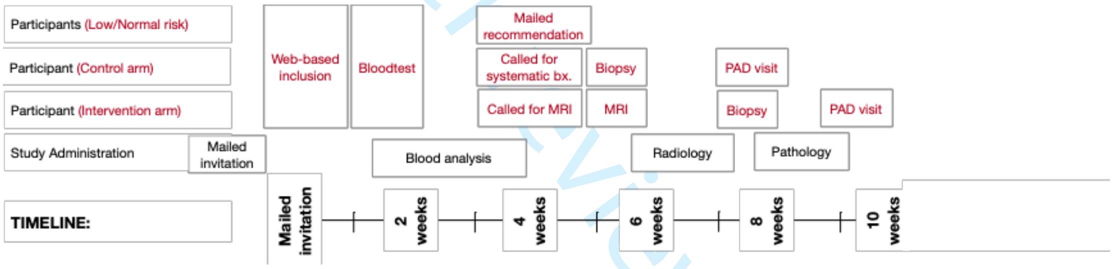

For peer review only

CONSORT 2010 checklist of information to include when reporting a randomised trial*

Section/Topic	Item No	Checklist item	Reported on page No
**Title and abstract**			
	1a	Identification as a randomised trial in the title	1
	1b	Structured summary of trial design, methods, results, and conclusions (for specific guidance see CONSORT for abstracts)	1
**Introduction**			
Background and objectives	2a	Scientific background and explanation of rationale	4-7
	2b	Specific objectives or hypotheses	8-9
**Methods**			
Trial design	3a	Description of trial design (such as parallel, factorial) including allocation ratio	9
	3b	Important changes to methods after trial commencement (such as eligibility criteria), with reasons	10
Participants	4a	Eligibility criteria for participants	10
	4b	Settings and locations where the data were collected	9
Interventions	5	The interventions for each group with sufficient details to allow replication, including how and when they were actually administered	10-13
Outcomes	6a	Completely defined pre-specified primary and secondary outcome measures, including how and when they were assessed	14
	6b	Any changes to trial outcomes after the trial commenced, with reasons	
Sample size	7a	How sample size was determined	15
	7b	When applicable, explanation of any interim analyses and stopping guidelines	16
Randomisation:			
Sequence generation	8a	Method used to generate the random allocation sequence	-
	8b	Type of randomisation; details of any restriction (such as blocking and block size)	10
Allocation concealment mechanism	9	Mechanism used to implement the random allocation sequence (such as sequentially numbered containers), describing any steps taken to conceal the sequence until interventions were assigned	10
Implementation	10	Who generated the random allocation sequence, who enrolled participants, and who assigned participants to interventions	
Blinding	11a	If done, who was blinded after assignment to interventions (for example, participants, care providers, those	-

For peer review only - http://bmjopen.bmj.com/site/about/guidelines.xhtml

		assessing outcomes) and how	
	11b	If relevant, description of the similarity of interventions	
Statistical methods	12a	Statistical methods used to compare groups for primary and secondary outcomes	Separate doc
	12b	Methods for additional analyses, such as subgroup analyses and adjusted analyses	Separate doc
**Results**			
Participant flow (a diagram is strongly recommended)	13a	For each group, the numbers of participants who were randomly assigned, received intended treatment, and were analysed for the primary outcome	
	13b	For each group, losses and exclusions after randomisation, together with reasons	
Recruitment	14a	Dates defining the periods of recruitment and follow-up	
	14b	Why the trial ended or was stopped	
Baseline data	15	A table showing baseline demographic and clinical characteristics for each group	
Numbers analysed	16	For each group, number of participants (denominator) included in each analysis and whether the analysis was by original assigned groups	
Outcomes and estimation	17a	For each primary and secondary outcome, results for each group, and the estimated effect size and its precision (such as 95% confidence interval)	
	17b	For binary outcomes, presentation of both absolute and relative effect sizes is recommended	
Ancillary analyses	18	Results of any other analyses performed, including subgroup analyses and adjusted analyses, distinguishing pre-specified from exploratory	
Harms	19	All important harms or unintended effects in each group (for specific guidance see CONSORT for harms)	
**Discussion**			
Limitations	20	Trial limitations, addressing sources of potential bias, imprecision, and, if relevant, multiplicity of analyses	
Generalisability	21	Generalisability (external validity, applicability) of the trial findings	
Interpretation	22	Interpretation consistent with results, balancing benefits and harms, and considering other relevant evidence	
**Other information**			
Registration	23	Registration number and name of trial registry	2
Protocol	24	Where the full trial protocol can be accessed, if available	1
Funding	25	Sources of funding and other support (such as supply of drugs), role of funders	18

*We strongly recommend reading this statement in conjunction with the CONSORT 2010 Explanation and Elaboration for important clarifications on all the items. If relevant, we also recommend reading CONSORT extensions for cluster randomised trials, non-inferiority and equivalence trials, non-pharmacological treatments, herbal interventions, and pragmatic trials. Additional extensions are forthcoming: for those and for up to date references relevant to this checklist, see www.consort-statement.org.

For peer review only

BMJ Open

Do a novel diagnostic pathway including blood-based risk-prediction and MRI-targeted biopsies outperform prostate cancer screening using prostate-specific antigen and systematic prostate biopsies? - The randomized, diagnostic study STHLM3MRI.

Journal:	*BMJ Open*
Manuscript ID	bmjopen-2018-027816.R1
Article Type:	Protocol
Date Submitted by the Author:	20-Feb-2019
Complete List of Authors:	Nordstrom, Tobias; Karolinska Inst, Dpt Medical Epidemiology and Biostatistics<br>Jäderling, Fredrik; Karolinska Institutet, Molecular Medicine and Surgery<br>Carlsson, Stefan; Karolinska Institutet<br>Aly, Markus; Karolinska Institutet,<br>Grönberg, H; Karolinska Institutet,<br>Eklund, Martin; Karolinska Institutet,
**Primary Subject Heading**:	Urology
Secondary Subject Heading:	Diagnostics
Keywords:	Prostate disease < UROLOGY, Magnetic resonance imaging < RADIOTHERAPY, Urological tumours < UROLOGY

SCHOLARONE™
Manuscripts

Title:

Do a novel diagnostic pathway including blood-based risk-prediction and MRI-targeted biopsies outperform prostate cancer screening using prostate-specific antigen and systematic prostate biopsies? - The randomized, diagnostic study STHLM3MRI.

Authors:

Tobias Nordström[a,b], MD PhD

Fredrik Jäderling[e,f], MD PhD

Stefan Carlsson[d,f], MD PhD

Markus Aly[a,d,f], MD PhD

Henrik Grönberg[a], MD PhD, Professor

Martin Eklund[a], PhD

[a]Department of Medical Epidemiology and Biostatistics, Karolinska Institutet, Sweden

[b]Department of Clinical Sciences at Danderyd Hospital, Karolinska Institutet, Sweden

[d]Department of Urology, Karolinska University Hospital Solna, Stockholm, Sweden

[e]Department of Diagnostic Radiology, Karolinska University Hospital, Stockholm,Sweden

[f]Department of Molecular Medicine and Surgery, Karolinska Institutet, Stockholm, Sweden

Correspondence to:

Tobias Nordström

Department of Medical Epidemiology and Biostatistics, Karolinska Institutet

S-171 77 Stockholm, Sweden

Email: tobias.nordstrom@ki.se

Phone: + 46 70 539 17 91

Keywords: Prostate cancer, Prostate neoplasm, biomarker, STHLM3, prostate biopsy, magnetic resonance imaging

For peer review only

1. *Abstract*

Introduction

Prostate cancer is a leading cause of cancer death among men in the Western world. Early detection of prostate cancer has been shown to decrease mortality, but has limitations with low specificity leading to unnecessary biopsies and over-diagnosis of low-risk cancers. The STHLM3 trial has paved the way for improved specificity in early detection of prostate cancer using the blood-based STHLM3 test for identifying men at increased risk of harbouring significant prostate cancer. Targeted prostate biopsies based on MRI images have been shown non-inferior sensitivity to detect significant prostate cancer and decrease the number of biopsies and non-significant cancers among men referred for prostate biopsy in clinical practice.

The strategy of the STHLM3-MRI projects is to study a diagnostic pathway including an improved blood-based test for identification of men with increased risk of prostate cancer and use of MRI to select men for diagnostic workup with targeted prostate biopsies.

Methods

This study compares prostate cancer detection using PSA and systematic biopsies with the improved pathway for prostate cancer detection using the STHLM3 test and targeted biopsies in a screening context. The study will recruit 10,000 participants during 1 June 2018- 1 June 2020 combining a paired and randomized design. Participants are grouped by PSA and Stockholm3 test level and men with Stockholm3≥11% or PSA ≥3ng/ml are randomized to systematic or MRI-targeted biopsies. This protocol follows SPIRIT guidelines. Endpoints include the number of detected prostate cancers, number of performed biopsy procedures and number of performed MRIs. Additional aims include to assess the health economic consequences and development of automated image-analysis.

Ethics and dissemination

The study has approval from the Regional Ethical Review Board in Stockholm (2017-1280/31). Study findings will be published in peer-review journals. Findings will also be disseminated by conference/departmental

For peer review only - http://bmjopen.bmj.com/site/about/guidelines.xhtml

presentations and by social/traditional media.

Registration details

ClinicalTrials.gov: NCT03377881

2. Strenghts and limitations of this study

- This is the first randomized study to examine the role of improved blood-based risk stratification used in sequence with MRI and targeted prostate biopsies in a screening-by-invitation context.
- The study examines the performance of the Stockholm3 test used together with MRI/Fusion technique compared with traditional PSA screening and will provide important data also on the performance of the Stockholm3 test or MRI/Fusion when used as standalone strategies.
- The study is performed at three study sites and uses centralized radiology and pathology.
- The study is limited to a Swedish screening population, the use of the Stockholm3 test as blood-based risk prediction and the used technology for MRI-targeted biopsies.

3. Trial identifier

ClinicalTrials.gov Identifier: NCT03377881

4. Introduction

4.1. Public health significance of prostate cancer

Prostate cancer is the most common cancer and the leading cause of cancer death among men in Sweden. In year 2011 over 10,000 men were diagnosed with prostate cancer and more than 2,500 died due to the disease, approximately 20% of these in the Stockholm region. Prostate cancer incidence rates in Sweden are now comparable to rates in countries that had an early introduction of PSA testing, while prostate cancer mortality rates in

For peer review only

Sweden are higher than in most other countries[1]. With over 90,000 prevalent cases, the health burden and the costs on the health care system are substantial. While a number of risk factors have been proposed for prevention of prostate cancer, including diet and occupational exposures, the only factors conclusively shown to increase risk of the disease are age, ethnicity and family history. Given the high prevalence of the cancer and limited opportunities for primary prevention, improved detection would reduce both procedure-related harm to men and economical cost in the healthcare system.

4.2. Early detection and treatment of prostate cancer: benefits and harms

The PSA test was first used to monitor disease progression in prostate cancer patients. The PSA test was taken up as a *de facto* screening test for prostate cancer in many countries, leading to rapid rises in prostate cancer incidence. The test characteristics for the PSA test in detecting prostate cancer are comparable to those for mammography for breast cancer screening, with a sensitivity of 72% and a specificity of 30-35% at a test threshold of 4 ng/ml[2]. However, a lower threshold of 3 ng/ml adopted in Sweden recently has led to increased sensitivity at the expense of reduced specificity. Recent analyses of PSA testing in the Stockholm area confirms these results showing that 46%, 68% and 77% of men 50-59, 60-69 and 70-79 years respectively have had at least one PSA test during a 9 years period[3].

Recent results from the large European Randomized Study of Screening for Prostate Cancer (ERSPC) including over 180,000 men provide increasing evidence that PSA screening has led to reduced mortality[4]. This report showed that PSA screening without digital rectal examination was associated with a 21% relative reduction in the death rate from prostate cancer at a median follow-up of 11 years, with an absolute reduction of about 7 prostate cancer deaths per 10,000 men screened. Estimations from the ERSPC trial (men aged 55-69) show that 1,048 men would need to be offered screening and an additional 37 would need to be managed to prevent one prostate-cancer death during a 10-year period, leading to a significant overtreatment of

For peer review only - http://bmjopen.bmj.com/site/about/guidelines.xhtml

indolent disease. The effectiveness of PSA testing was more marked at the Göteborg site of the ERSPC trial, with a risk reduction of 44% over 14 years in men aged 50-64[5]. This effect size is larger than that observed for mammographic screening for breast cancer and faecal occult blood testing for colorectal cancer.

However, using traditional systematic biopsies for diagnosis, approximately half of diagnosed cancers are low-risk tumours using the same main cut-off for biopsy as the ERSPC trial (PSA=3ng/ml) [6,7]. It has been shown that men with low-risk tumours treated without curative intent have the same survival as men in the background population[8], illustrating the large proportion of over-diagnosed cancers[9].

The STHLM3 study has shown a way to improve identification of men at increased risk of significant prostate cancer. Using the STHLM3 test, 32% of the prostate biopsies may be saved while not decreasing the sensitivity to high-grade disease (defined as Gleason Score ≥7) and simultaneously decreasing the number of low-grade tumours (Gleason Score ≤6) by 17%, thus decreasing overdiagnosis[7].

4.3. Traditional evaluation of men with increased risk of prostate cancer

Men at increased risk of prostate cancer - commonly estimated using PSA and palpatory findings - are traditionally assessed using systematic prostate biopsies. The procedure is performed under local anaesthesia using antibiotic prophylaxis and includes 10-12 cores taken from predefined areas of the peripheral zone of the gland as visualized by endorectal ultrasound. While the biopsies systematically covers the prostatic gland rather than targeting a lesion, and non-lethal tumours are common, the risk of over-diagnosis (i.e. detection of non-significant tumours) is high [9]. The risk of non-representative biopsy findings result in underestimation of tumour grade compared with subsequent prostatectomy in up to 40% of men undergoing surgery[10]. The risk of severe post-biopsy infection has increased to 1-2% with increasing frequency of antibiotic resistance, further illustrating the need both to increase precision and decrease the number of performed biopsies[11]. Since screening using PSA and systematic prostate biopsies have been shown to

For peer review only - http://bmjopen.bmj.com/site/about/guidelines.xhtml

decrease prostate cancer mortality, it is reasonable to use this strategy as comparator for novel diagnostic strategies[4-5].

4.4. Multi-parametric Magnetic Resonance Imaging (mpMRI) for detection of prostate cancer

Multi-parametric magnetic resonance imaging (mpMRI) incorporating anatomical and functional imaging has now been validated as a means of detecting and characterizing prostate tumours and can aid in risk stratification and treatment selection. The European Society of Urogenital Radiology (ESUR) in 2012 established the Prostate Imaging Reporting and Data System (PI-RADS) guidelines aimed at standardizing the acquisition, interpretation and reporting of prostate mpMRI. Consensus on an updated version (PI-RADS v2) have recently been published, outlining aspects of both interpretation and the technical execution[12-14]. Use of the revised PI-RADS provides moderately reproducible MR imaging scores for detection of clinically relevant disease[15]. Using MP-MRI to triage men might allow 27% of patients avoid a primary biopsy and diagnosis fewer clinically insignificant cancers. If subsequent TRUS-biopsies were directed by MP-MRI findings, up to 18% more cases of clinically significant cancer might be detected compared with the standard pathway of TRUS-biopsy for all[16].

In summary, PI-RADS recommends to use 3T or 1.5T machines, including T2- and T1-weighted sequences together with diffusion weighted images (DWI). Currently, the added value of dynamic contrast is not firmly established regarding tumour detection. At this time, there is no consensus among experts concerning the potential benefits of the use of endorectal coils for cancer detection. It has been suggested that the prevalence of suspicious lesions on MRI in men with clinical suspicion of prostate cancer is approximately 60% [17].

4.5. Targeted prostate biopsies guided by fusion technology

Targeted biopsies of the prostate consist of imaging (MRI) detecting significant tumours and a biopsy procedure where biopsies are targeted to the tumour using various devices for guidance[18]. While traditional endorectal ultrasound poorly identifies tumours, direction of biopsy needles can be

For peer review only - http://bmjopen.bmj.com/site/about/guidelines.xhtml

performed in various ways. Cognitive or soft fusion is based on skilled urologists/radiologists interpreting the MRI images and directing needles solely based on the ultrasound images. The disadvantages of cognitive fusion lie in the potential for human error when attempting to mentally fuse the MRI with TRUS while aiming for cancers that are often <1 cm in diameter and the inability to track the location of each biopsy site. Hard fusion enables proper fusion of MRI information on the ultrasound image, possibly increasing precision.

Despite methodological flaws, a number of studies have investigated the value of fusion biopsies, primarily using non-randomized designs and non-screening populations[19]. In 2018, Kasivisvanathan et al provided high quality evidence for men referred for prostate biopsy and showed that MRI/target biopsies are non-inferior for detection of significant cancer and decreases the number of in-significant cancers and number of biopsies as compared with systematic biopsies[20].

The proportion of men upgraded when comparing specimen from targeted biopsies and subsequent prostatectomy have been shown to be very low (<5%) when using targeted biopsies[21], increasing the proportion of men where treatment decisions are based on valid risk estimations.

4.6. Improving the diagnostic pathway for prostate cancer detection

The current diagnostic pathway for prostate cancer detection is characterized by several challenging hallmarks. First, testing with PSA is frequent also in men not benefitting from testing due to low PSA levels or high age[3]. Second, the currently used test for detection (PSA) lacks in specificity, resulting in frequent over-diagnosis[22,23]. Third, systematic biopsies shows high frequencies of benign tests, over-diagnosis, up-grading at prostatectomy, and risk of infectious complications[7,24]. Further, PSA testing increases with educational length and men with long education are more likely to have a prostate biopsy after an increased PSA value. These differences may contribute to the worse prostate cancer outcomes observed among men with lower socioeconomic status[25].

For peer review only - http://bmjopen.bmj.com/site/about/guidelines.xhtml

The STHLM3 test offers improved disease detection[7]. To further decrease over-detection, improve disease classification and spare men of test-related harm, prostate biopsy practices need to be improved. We hypothesize that an improved pathway for prostate cancer detection including a better blood-based screening test, improved selection to biopsy based on MRI findings and targeted biopsies guided by MRI/ultrasound fusion would dramatically decrease the number of biopsy procedures, overdiagnosis and improve treatment decisions.

5. Methods

5.1. Hypotheses

5.1.1. Primary hypotheses

The below hypothesis is posed for men in screening-by-invitation context:

A diagnostic pathway using the Stockholm3 test to select men for further workup using MRI followed by targeted biopsies and systematic biopsies (S3M-MR-TBx/SBx) has non-inferior sensitivity for detecting clinically significant cancer (ISUP grade group ≥ 2) and shows superior specificity (reduction in number of performed biopsy procedures and detected ISUP 1 tumours) compared to a diagnostic pathway using systematic biopsies in men with PSA ≥3 ng/ml (PSA-SBx).

5.1.2. Additional hypotheses

1. As compared with performing systematic biopsies for men with elevated risk of prostate cancer in prostate cancer screening, targeted prostate biopsies performed with MRI/Fusion technique with or without addition of systematic biopsies has non-inferior sensitivity for detecting clinically significant cancer (ISUP grade group ≥ 2) and reduces the number of performed biopsy procedures.
2. A diagnostic pathway using the Stockholm3 test to select men for further workup using MRI followed by ONLY targeted biopsies (S3M-

For peer review only

MR-TBx) has non-inferior sensitivity for detecting clinically significant cancer (ISUP grade group ≥ 2) and reduces the number of performed biopsy procedures compared to a diagnostic pathway using systematic biopsies in men with PSA ≥3 ng/ml (PSA-SBx).

3. Adding prostate volume as parameter in the diagnostic pathway with Stockholm3 test and MRI/Fusion biopsies improves model precision.
4. A diagnostic pathway with Stockholm3 followed by MRI and targeted biopsies has non-inferior sensitivity for detecting clinically significant cancer (ISUP grade group ≥ 2) and reduces the number of MRI examinations and performed biopsies compared to a diagnostic pathway using PSA ≥3 ng/ml followed by MRI and targeted biopsies.
5. SBx in the MRI arm has superior sensitive than SBx in the non-MRI arm (due to cognitive fusion).
6. Biopsy compliance is higher after biopsy is recommended based on MRI compared to recommended without MRI.
7. A diagnostic pathway using the Stockholm3 test to select men for further workup using MRI and targeted biopsies (S3M+TBx) shows better health economy (positive ICER) compared to a diagnostic pathway using systematic biopsies in men with PSA ≥3 ng/ml (PSA+SBx).

5.2. Aims

To compare a diagnostic pathway using the Stockholm3 test (S3M ≥ 11%) to select men for further workup using MRI (PI-RADS ≥ 3) and targeted biopsies (S3M+TBx) to a diagnostic pathway using systematic biopsies in men with PSA ≥3 ng/ml (PSA+SBx) with respect to number of diagnosed clinically significant cancer (ISUP grade group ≥ 2) and number of performed biopsies. Additional aims corresponding to hypotheses 2-8 above will be assessed.

5.3. Study design

For peer review only - http://bmjopen.bmj.com/site/about/guidelines.xhtml

STHLM3-MR Phase 2 is a study combining a paired and a randomized design (Figure 1). The study will follow the following outline: Participants will be invited by mail. All participants will undergo a blood-test, including PSA and the STHLM3 test. Men with an elevated PSA ≥3 ng/ml *or* PSA ≥1.5ng/ml and S3M>11% will be randomized to either traditional prostate biopsies or MR with targeted biopsies on MR lesions.

5.4. Participants, interventions and outcomes

5.4.1. Study setting

This is a screening-by-invitation study including one study administrative centre, two radiological sites and three urological sites where data will be collected.

Participating urological centres

Department of Urology, Capio St Görans Hospital: dr Henrik Grönberg

Uroclinic, Sophiahemmet, Stockholm; dr Olof Jansson

Odenplans läkarhus; dr Magnus Annerstedt

5.4.2. Eligibility criteria

Inclusion criteria

Men age 50-74 years without prior diagnosis of prostate cancer (ICD-9 C61).

Permanent postal address in Stockholm

Not a previous participant in the Stockholm3 study (2012-2014)

Exclusion criterias

Severe illnesses such as metastatic cancers, severe cardio-vascular disease or dementia

Contraindications for magnetic resonance imaging (MRI) eg pacemaker, magnetic cerebral clips, cochlear implants or severe claustrophobia.

Men with a previous prostate biopsy the preceding 60 days before invitation.

For peer review only

5.4.3. Randomization

Randomization is performed 2:3 between control arm and experimental arm. Randomization will be performed will be performed using stratification on disease risk [6 strata]. Disease risk is assessed using the Stockholm3 test. Test are discordant if PSA is negative and Stockholm3 positive or vice versa.

Four allocation lists [high/low risk vs discordant/concordant tests] have been created with the sequence [control arm, control arm, experimental arm, experimental arm, experimental arm]. Participants are first allocated to corresponding list, and then allocated to study arm according to the order in which they participate. The allocation sequence is blinded for the study investigators and handled by the study database administrator (A Björklund).

In order to enhance resource usage, men are allocated to the study sites according to local availability of biopsy procedure slots.

5.4.4 Interventions

Blood sampling

Participating men undergo blood-sampling with analysis of PSA and the Stockholm3 test at Karolinska University Laboratory.

For the main analysis, the Stockholm3 test include clinical data as answered when consenting participation (previous biopsy, age, finasteride medication, relatives with prostate cancer); single nucleotide polymorphisms and measurements of protein levels (MSMB, MIC1, PSA, fPSA, hK2)[7]. For secondary analyses, clinical information on DRE and prostate volume is included.

Definition of EXPERIMENTAL ARM

Men randomized to the experimental arm undergoes MRI. If suspicious lesions are found, the participant undergoes targeted biopsies using Fusion technology *followed by systematic biopsies.*

For peer review only - http://bmjopen.bmj.com/site/about/guidelines.xhtml

332 Men without lesions are excepted from further intervention and receives notification on recommendation for follow-up. Technology and process are described below.

335 Men with a Stockholm3 risk ≥25% and no suspicious lesion on MRI will undergo systematic biopsies.

337 ***Definition of CONTROL ARM***

338 Men randomized to the control arm undergoes systematic biopsies as defined below.

340 ### 5.4.5 Technology

341 ***Cut-offs for performing the STHLM3 test***

342 The STHLM3 test will be performed for men with a PSA ≥ 1.5 ng/ml

343 ***Cut-offs for entering randomization***

344 Participants with PSA ≥ 3.0 ng/ml or STHLM3-test ≥ 11% risk of Gleason Score ≥7 cancer will be randomized and offered to undergo either MR or systematic biopsies (See Process description).

347 ***MRI technology***

348 *Location and MRI equipment*

349 Capio St Görans Hospital: General Electric, Architect, 3T
350 Globen Unilabs Healthcare: Siemens Magnetom Aera 1.5T

351 *Patient preparations*

352 Refraining from sexual activity with ejaculation 3 days prior to examination
353 Fasting patient 6 h
354 Minimal preparation enema prior to examination
355 Antispasmodic agent (Glucagon) just before the examination

356 *MRI Protocol*

357 A short (14 minutes) MRI protocol will be used. A detailed description is available. Briefly, the protocol includes: T2w images axial, sagittal, coronal;

For peer review only - http://bmjopen.bmj.com/site/about/guidelines.xhtml

Diffusion weighted imaging b0 and b1000 with ADC and a synthetic b1500 limited to the prostate location; No endorectal coil will be used.

MRI Interpretation

MRI interpretation is centralized to Capio St Görans hospital and is performed according to PIRAD v2.0 for examinations without adequate perfusion studies. Dr Fredrik Jäderling is responsible for MRI interpretation. Dr Jäderling or 1-2 other, experienced radiologists at his department performs all MRI interpretations.

PI-RADS v2 (“Assessment without adequate dynamic contrast enhanced imaging”) will be used, with a 1-5 grade scale of suspicious lesions (1= clinically significant cancer is highly unlikely to be present, 5= clinically significant cancer is highly likely to be present).

During the study period participating radiologist will have access to updated histology results of fusion biopsies to be able to adjust their MRI reading according to tumour detection rates for different PIRAD diagnoses as defined above.

Fusion biopsy technology

Brand/models

BK Medical (BK Ultrasound ; www.bkultrasound.com/bk-medical/fusion)

The BK Medical fusion system is the only fusion device compatible with BK Medicals ultrasound devices, used by the urology departments participating in the study. The system represents a second generation ultrasound system with integrated MRI Fusion. MRI data is imported through HIPAA-compliant PACS connection with the local radiology department.

Definition of targeted biopsies

Using MRI data with pre-marked borders of the prostate and tumor, fusion of MRI images and ultrasound images are performed bedside. Using local anesthetic and antibiotic prophylaxis, lesions are according to below. Targeted biopsies are always combined with systematic biopsies.

For peer review only - http://bmjopen.bmj.com/site/about/guidelines.xhtml

Biopsy procedure for targeted biopsies

PI-RADS≥3: 3-4 targeted biopsies on marked lesions + systematic biopsies.

Large diffuse lesions or poor image quality: Systematic biopsies including lesion.

No PI-RADS≥3, diffuse lesions and at least acceptable image quality: No biopsies are performed.

In larger lesions in PI-RADS category 3 and 5, areas within the lesion with the lowest ADC value ("Target-within-target") will be targeted with the first biopsy taken from the lesion, to evaluate the additional value regarding tumor staging.

Definition of systematic biopsies

10-12 systematic biopies are taken from the peripheral zone as previously described in STLHLM3 and the National Guidelines. Extra biopsies are allowed from additional sites visible on ultrasound or according to palpatory findings. In summary, systematic biopsies are performed in the peripheral zone as 4 lateral and para-median biopsies on the left and right side, in the base and mid part of the gland. In the apical third of the gland one lateral left and right biopsy is performed.

Pathology

Pathology is centralized to Unilabs/Capio St Görans hospital. Dr Axel Glaessgen is responsible for the integrity of analyzes of pathological specimen. 2-3 uro-pathologists at dr Glaessgens department assesses all pathological specimen with intermittent cross-validation between them. Pathology preparation and reporting follow ISUP 2014 guidelines.

The pathology preparation is done by Unilabs as part of the normal clinical routine. Biopsy specimens are analyzed according to local practice.

Localisation of biopsies in the prostate are described using Swedish National Guideline nomenclature (A1-4; B1-4; C1-4; anterior/posterior).

For peer review only

For peer review only - http://bmjopen.bmj.com/site/about/guidelines.xhtml

Gleason Score, mm cancer and % Gleason 4 is reported on each needle specimen.

Pathologist notes results in the usual way in the laboratory system. The result of the pathological analysis is submitted in accordance to existing clinical routines to the referring urologist. A copy of the result is delivered to the study administration.

5.4.4. Outcomes

There are three co-primary endpoints in this trial: (i) Number of diagnosed ISUP grade group ≥ 2 cancers; (ii) Number of diagnosed ISUP grade group 1 cancers; (iii) Number of performed biopsies.

5.4.5. Follow-up

Main study outcomes are assessed after prostate biopsy procedures. Additional participant data will be secured in the following circumstances:

No suspicious lesion on MRI

Men in the experimental arm without suspicious lesions on MRI will be informed and recommended follow-up by the responsible, local urologist. After additional ethical application, the co-investogators might initiate retrospective follow-up of these participants.

Men with diagnosed prostate cancer

Participants with prostate cancer diagnosed on biopsy within the study will be followed up after the biopsy to secure data on the following: Treatment modality (Active Surveillance, Surgery, Radiation); Treatment lead-time and site; Pathological report after surgery (positive margins, T-stage, etc). Data will be assessed through medical records intermittently.

5.5. Serious adverse events

Study nurse will monitor serious adverse events after the prostate biopsy procedures. To ensure this, the study nurse will follow this check medical journals for hospitalization within 1 week after the biopsy procedure in the

For peer review only - http://bmjopen.bmj.com/site/about/guidelines.xhtml

journal systems Take Care and Cosmic (covering the main part of hospitals in Stockholm region). This will be initiated as individual biopsy results are registered at the study administration. Results will be provided to the Data Safety and Monitoring Board.

5.6. Participant timeline

Figure 2 illustrates the approximate timeline for participating men in STHLM3MRI Main Study.

5.7. Sample size

STHLM3-MR/Fusion Phase 2 will invite 25,000 men and aim to include 10,000 participants. We anticipate to perform 1,039 biopsy procedures altogether. Inclusion will continue until complete data on 415 men in the control arm (SBx) and 623 men in the experimental arm (MR-TBx-SBx).

Basic data and assumptions used in the sample size calculations

We used data from the STHLM3 trial for sample size calculations [7]. In this data, 18% of men with PSA ≥ 3 had a clinically significant prostate cancer when biopsied with SBx. We further noted that rTPR=1.45 for clinically significant prostate cancer comparing MRI+TBx with SBx based on the results from the PRECISION randomized trial [20]. However, we will for sample size calculations use rTPR=1.25 for MRI+TBx vs. SBx as a more conservative estimate. We set the non-inferiority delta to 4 percentage points for demonstrating noninferiority with respect to sensitivity of clinically significant prostate cancer. We set the alpha to 5%.

Primary contrast

Simulating 1000 trials (by bootstrapping from the STHLM3 data) under the assumptions outlined in the preceding section 303 men need to biopsied in the SBx arm based on PSA ≥ 3 to have 80% power to demonstrate non-inferior sensitivity of S3M+MRI+TBx compared with PSA+SBx. This means that at least **415** men need to be biopsied in the SBx arm (since some men are not randomized based on PSA ≥ 3 but on S3M ≥ 11%) and, consequently,

For peer review only - http://bmjopen.bmj.com/site/about/guidelines.xhtml

623 to the MRI arm (because of the 2:3 randomization). Total number of men undergoing workup according to protocol (SBx in the no MRI arm and MRI and TBx if Pi-RADS ≥ 3 in the MRI arm) is thus 1038. Assuming 20% dropout, 1300 men need to be randomized. These numbers give 80% power to detect a modest 17% reduction in biopsies between the two strategies.

5.8. Recruitment and Process Description

The STHLM3-MR Phase 2 will use existing solutions developed and optimized in the previous studies STHLM3 and STHLM3-MR Phase 1 where all major components of the process have been tested. First, participants will follow the *paired design study process* where inclusion, blood-test and delivery of recommendation letter is performed. Men with increased risk of high-grade prostate cancer then enter the *randomized study process*, where extended work-up including biopsies are performed.

5.9. Data Collection, management, analysis

5.9.1. Data collection

Primary data sources are

i. clinical variables collected from laboratory referral
ii. biopsy referrals and reports
iii. pathology reports
iv. MRI reports
v. blood concentrations of kallikreins, MSMB, MIC1, SNPs

Collection of i. – iv. is performed by study nurses (C Cavalli-Björkman) on a weekly basis from participating urology sites, participating radiologists. For v., this is digitally transferred from Karolinska University Laboratory.

5.9.2. Data management

Data is collected, entered, coded and stored at Department of Medical Epidemiology and Biostatistics, Karolinska Institutet. Data is entered by Study Nurse using predefined database sheets developed in STHLM3MRI Phase 1. This is blinded from study co-investigators and data is stored at the department under supervision by the study database administrator (SDA,

For peer review only - http://bmjopen.bmj.com/site/about/guidelines.xhtml

Astrid Björklund). Any extraction of study data is performed by the SDA after approval of PI Tobias Nordström.

5.9.3. Data analysis

Analysis of data is described in the Statistical Analysis Plan (SAP).

5.9.4. Auditing and Monitoring

A Data Safety and Monitoring Board (DSMB) is assembled and consist of dr Hans Garmo (Statistician), prof Ola Bratt (Urology) and prof Holmberg (Urology/Study Design). The DSMB audits protocol and process descriptions and one interim data extraction performed by the study database administrator after 10% (100 men) have completed the control or experimental arms. The co-investigators are blinded to the interim data and analysis results. The work of the DSMB is regulated in the DSMB Charter.

5.10. Patient and Public Involvement

The research question and outcome measures were designed to improve prostate cancer diagnostics. This includes optimizing prostate biopsies and decreasing over-detection, both associated with morbidity. Patient organisations were informed on the results from the STHLM3MRI Phase 1 study. Patients were not involved in recruitment of the study. Results will be disseminated to participants through common and scientific channels.

6. Ethics and dissemination

6.1. Research ethics approval

The study has approval from the Regional Ethical Review Board in Stockholm (2017-1280/31).

6.2. Consent

Participant consent is secured when the participant is included to the study at www.kliniskastudier.se. This includes secure identification using Mobilt

For peer review only

BankID. Additional approval on use of biological specimen data is collected on the biopsy referral.

6.3. Confidentiality

Study data is collected and stored at Department of Medical Epidemiology and Biostatistics, Karolinska Institutet using secure Oracle servers. All data extractions are made by database administrator and are anonymized (personal id number is removed) before dissemination to researchers.

6.4. Dissemination

Analyses results on the posed aims will be submitted for peer-reviewed publication and submitted for presentation at scientific congress. Communication of the results will be made to patient organizations (Prostatacancerförbundet) and non-scientific channels. No use of professional writers is planned.

The study protocol is made publicly available through clinicaltrials.gov.

6.5. Data Sharing Statement

Anonymized, individual participant data that underlie the results reported in this article, after deidentification (text, tables, figures and appendices) will be available for data sharing. Proposals may be submitted up to 36 months following article publication. Data will be shared with investigators whose proposed use of the data has been approved by an independent review committee identified for this purpose.

7. Declarations of interest

Henrik Grönberg has five prostate cancer diagnostic related patents pending, has patent applications licensed to Thermo Fisher Scientific, and might receive royalties from sales related to these patents. Martin Eklund is named on four of these five patent applications. Karolinska Institutet collaborates with Thermo Fisher Scientific in developing the technology for the Stockholm3 test.

For peer review only - http://bmjopen.bmj.com/site/about/guidelines.xhtml

8. Contributions

TN was the Principal investigator. TN, HG, ME, SC and MA designed the study. ME and TN interpreted preliminary data. FJ designed MRI protocols and collected data.

We thank participants, study organizers, participating researchers and clinicians, and patient advisers for their contributions to the STHLM3MRI project.

9. Funding statement

Funding was provided by the Swedish Cancer Society, (Cancerfonden), the Swedish Research Council (Vetenskapsrådet), Swedish Research Council for Health Working Life and Welfare (FORTE), The Strategic Research Programme on Cancer (StratCan), Karolinska Institutet, Swedish e-Science Research Center (SeRC) and Stockholm City Council (SLL). The STHLM3 study is a part of the Linnaeus Center CRISP ‘‘Predication and prevention of breast and prostate cancer’’ funded by the Swedish Research Council.

10. Figure legends

Figure 1: Study design overview STHLM3MRI Main Study

Figure 2: Timeline overview for study participants in STHLM3MRI Main Study

11. References

1 Ferlay J, Soerjomataram I, Dikshit R, *et al.* Cancer incidence and mortality worldwide: Sources, methods and major patterns in GLOBOCAN 2012. *Int J Cancer* 2014;:n/a–n/a. doi:10.1002/ijc.29210

2 Mistry K, Cable G. Meta-analysis of prostate-specific antigen and digital rectal examination as screening tests for prostate carcinoma. *J Am Board Fam Pract* 2003;**16**:95–101.

3 Nordström T, Aly M, Clements MS, *et al.* Prostate-specific antigen (PSA) testing is prevalent and increasing in Stockholm County, Sweden, Despite no recommendations for PSA screening: results from a

For peer review only - http://bmjopen.bmj.com/site/about/guidelines.xhtml

population-based study, 2003-2011. *Eur Urol* 2013;**63**:419–25. doi:10.1016/j.eururo.2012.10.001

4 Schröder FH, Hugosson J, Roobol MJ, *et al.* Prostate-cancer mortality at 11 years of follow-up. *N Engl J Med* 2012;**366**:981–90. doi:10.1056/NEJMoa1113135

5 Hugosson J, Carlsson S, Aus G, *et al.* Mortality results from the Göteborg randomised population-based prostate-cancer screening trial. *Lancet Oncol* 2010;**11**:725–32. doi:10.1016/S1470-2045(10)70146-7

6 Nationell kvalitetsrapport för diagnosår 2012. Regionala Cancercentrum i Samverkan 2013. http://npcr.se/wp-content/uploads/2013/04/20131121-NPCR-Rapport-2012.pdf

7 Grönberg H, Adolfsson J, Aly M, *et al.* Prostate cancer screening in men aged 50-69 years (STHLM3): a prospective population-based diagnostic study. *Lancet Oncol* 2015;**16**:1667–76.

8 Rider JR, Sandin F, Andrén O, *et al.* Long-term outcomes among noncuratively treated men according to prostate cancer risk category in a nationwide, population-based study. *Eur Urol* 2013;**63**:88–96. doi:10.1016/j.eururo.2012.08.001

9 Loeb S, Bjurlin MA, Nicholson J, *et al.* Overdiagnosis and overtreatment of prostate cancer. *Eur Urol* 2014;**65**:1046–55. doi:10.1016/j.eururo.2013.12.062

10 Soares R, Di Benedetto A, Dovey Z, *et al.* Minimum 5-year follow-up of 1138 consecutive laparoscopic radical prostatectomies. *BJU Int* 2015;**115**:546–53. doi:10.1111/bju.12887

11 Aly M, Dyrdak R, Nordström T, *et al.* Rapid increase in multidrug-resistant enteric bacilli blood stream infection after prostate biopsy-A 10-year population-based cohort study. *Prostate* 2015;**75**:947–56. doi:10.1002/pros.22979

12 Barrett T, Turkbey B, Choyke PL. PI-RADS version 2: what you need to know. *Clin Radiol* 2015;**70**:1165–76. doi:10.1016/j.crad.2015.06.093

13 Barentsz JO, Weinreb JC, Verma S, *et al.* Synopsis of the PI-RADS v2 Guidelines for Multiparametric Prostate Magnetic Resonance Imaging and Recommendations for Use. *Eur Urol* 2015;**69**:41–9. doi:10.1016/j.eururo.2015.08.038

14 Weinreb JC, Barentsz JO, Choyke PL, *et al.* PI-RADS Prostate Imaging - Reporting and Data System: 2015, Version 2. *Eur Urol* 2015;**69**:16–40. doi:10.1016/j.eururo.2015.08.052

15 Muller BG, Shih JH, Sankineni S, *et al.* Prostate Cancer: Interobserver Agreement and Accuracy with the Revised Prostate Imaging Reporting

For peer review only

For peer review only - http://bmjopen.bmj.com/site/about/guidelines.xhtml

and Data System at Multiparametric MR Imaging. *Radiology* 2015;**277**:741–50. doi:10.1148/radiol.2015142818

16 Ahmed HU, El-Shater Bosaily A, Brown LC, *et al.* Diagnostic accuracy of multi-parametric MRI and TRUS biopsy in prostate cancer (PROMIS): a paired validating confirmatory study. *Lancet* Published Online First: 19 January 2017. doi:10.1016/S0140-6736(16)32401-1

17 Moore CM, Robertson NL, Arsanious N, *et al.* Image-guided prostate biopsy using magnetic resonance imaging-derived targets: a systematic review. *Eur Urol* 2013;**63**:125–40. doi:10.1016/j.eururo.2012.06.004

18 Sonn GA, Margolis DJ, Marks LS. Target detection: magnetic resonance imaging-ultrasound fusion-guided prostate biopsy. *Urol Oncol* 2014;**32**:903–11. doi:10.1016/j.urolonc.2013.08.006

19 Schoots IG, Roobol MJ, Nieboer D, *et al.* Magnetic Resonance Imaging-targeted Biopsy May Enhance the Diagnostic Accuracy of Significant Prostate Cancer Detection Compared to Standard Transrectal Ultrasound-guided Biopsy: A Systematic Review and Meta-analysis. *Eur Urol* 2014;**68**:438–50. doi:10.1016/j.eururo.2014.11.037

20 Kasivisvanathan V, Rannikko AS, Borghi M, *et al.* MRI-Targeted or Standard Biopsy for Prostate-Cancer Diagnosis. *N Engl J Med* 2018;:NEJMoa1801993. doi:10.1056/NEJMoa1801993

21 Baco E, Ukimura O, Rud E, *et al.* Magnetic resonance imaging-transectal ultrasound image-fusion biopsies accurately characterize the index tumor: correlation with step-sectioned radical prostatectomy specimens in 135 patients. *Eur Urol* 2015;**67**:787–94. doi:10.1016/j.eururo.2014.08.077

22 Arnsrud Godtman R, Holmberg E, Lilja H, *et al.* Opportunistic Testing Versus Organized Prostate-specific Antigen Screening: Outcome After 18 Years in the Göteborg Randomized Population-based Prostate Cancer Screening Trial. *Eur Urol* 2014;**68**:354–60. doi:10.1016/j.eururo.2014.12.006

23 Thompson IM, Pauler DK, Goodman PJ, *et al.* Prevalence of prostate cancer among men with a prostate-specific antigen level. *N Engl J Med* 2004;**350**:2239–46. doi:10.1056/NEJMoa031918

24 Loeb S, Vellekoop A, Ahmed HU, *et al.* Systematic review of complications of prostate biopsy. *Eur Urol* 2013;**64**:876–92. doi:10.1016/j.eururo.2013.05.049

25 Nordström T, Bratt O, Örtegren J, *et al.* A population-based study on the association between educational length, prostate-specific antigen testing and use of prostate biopsies. *Scand J Urol* 2016;**50**:104–9. doi:10.3109/21681805.2015.1113200

For peer review only

666

For peer review only

For peer review only - http://bmjopen.bmj.com/site/about/guidelines.xhtml

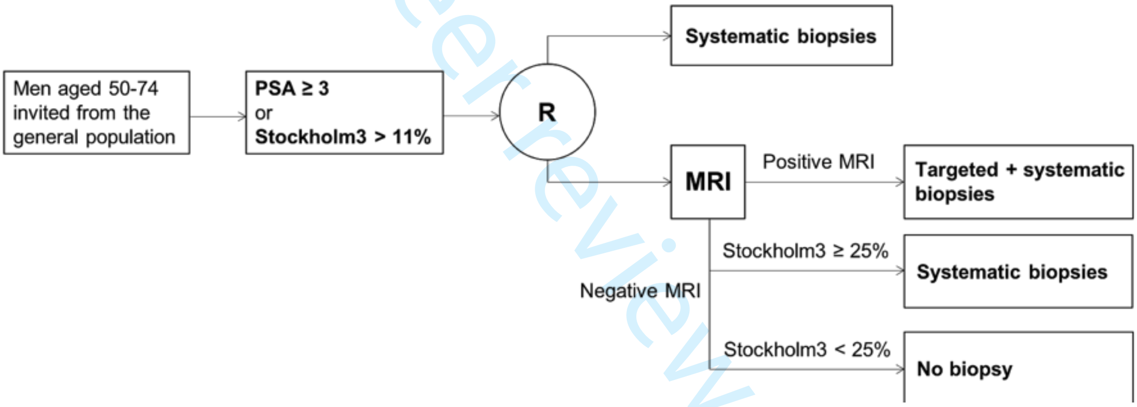

For peer review only

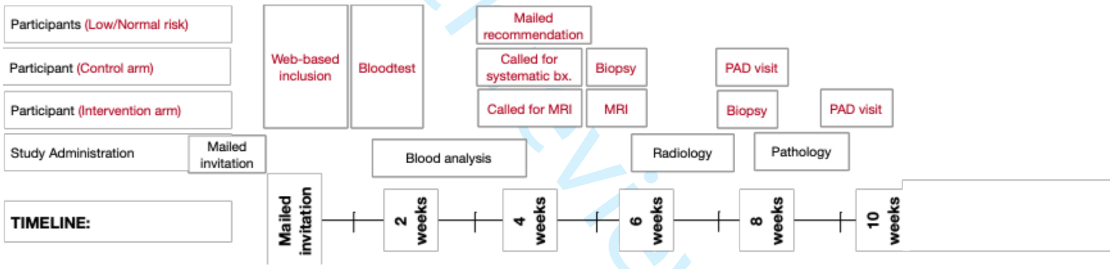
Participants (Low/Normal risk)
Participant (Control arm)
Participant (Intervention arm)
Study Administration
Mailed invitation
Web-based inclusion
Bloodtest
Mailed recommendation
Called for systematic bx.
Biopsy
Called for MRI
MRI
Blood analysis
PAD visit
Biopsy
PAD visit
Radiology
Pathology
TIMELINE:
Mailed invitation
2 weeks
4 weeks
6 weeks
8 weeks
10 weeks

CONSORT 2010 checklist of information to include when reporting a randomised trial*

Section/Topic	Item No	Checklist item	Reported on page No
**Title and abstract**			
	1a	Identification as a randomised trial in the title	1
	1b	Structured summary of trial design, methods, results, and conclusions (for specific guidance see CONSORT for abstracts)	1
**Introduction**			
Background and objectives	2a	Scientific background and explanation of rationale	4-7
	2b	Specific objectives or hypotheses	8-9
**Methods**			
Trial design	3a	Description of trial design (such as parallel, factorial) including allocation ratio	9
	3b	Important changes to methods after trial commencement (such as eligibility criteria), with reasons	10
Participants	4a	Eligibility criteria for participants	10
	4b	Settings and locations where the data were collected	9
Interventions	5	The interventions for each group with sufficient details to allow replication, including how and when they were actually administered	10-13
Outcomes	6a	Completely defined pre-specified primary and secondary outcome measures, including how and when they were assessed	14
	6b	Any changes to trial outcomes after the trial commenced, with reasons	
Sample size	7a	How sample size was determined	15
	7b	When applicable, explanation of any interim analyses and stopping guidelines	16
Randomisation:			
Sequence generation	8a	Method used to generate the random allocation sequence	-
	8b	Type of randomisation; details of any restriction (such as blocking and block size)	10
Allocation concealment mechanism	9	Mechanism used to implement the random allocation sequence (such as sequentially numbered containers), describing any steps taken to conceal the sequence until interventions were assigned	10
Implementation	10	Who generated the random allocation sequence, who enrolled participants, and who assigned participants to interventions	
Blinding	11a	If done, who was blinded after assignment to interventions (for example, participants, care providers, those	-

For peer review only - http://bmjopen.bmj.com/site/about/guidelines.xhtml

		assessing outcomes) and how	
	11b	If relevant, description of the similarity of interventions	
Statistical methods	12a	Statistical methods used to compare groups for primary and secondary outcomes	Separate doc
	12b	Methods for additional analyses, such as subgroup analyses and adjusted analyses	Separate doc
**Results**			
Participant flow (a diagram is strongly recommended)	13a	For each group, the numbers of participants who were randomly assigned, received intended treatment, and were analysed for the primary outcome	
	13b	For each group, losses and exclusions after randomisation, together with reasons	
Recruitment	14a	Dates defining the periods of recruitment and follow-up	
	14b	Why the trial ended or was stopped	
Baseline data	15	A table showing baseline demographic and clinical characteristics for each group	
Numbers analysed	16	For each group, number of participants (denominator) included in each analysis and whether the analysis was by original assigned groups	
Outcomes and estimation	17a	For each primary and secondary outcome, results for each group, and the estimated effect size and its precision (such as 95% confidence interval)	
	17b	For binary outcomes, presentation of both absolute and relative effect sizes is recommended	
Ancillary analyses	18	Results of any other analyses performed, including subgroup analyses and adjusted analyses, distinguishing pre-specified from exploratory	
Harms	19	All important harms or unintended effects in each group (for specific guidance see CONSORT for harms)	
**Discussion**			
Limitations	20	Trial limitations, addressing sources of potential bias, imprecision, and, if relevant, multiplicity of analyses	
Generalisability	21	Generalisability (external validity, applicability) of the trial findings	
Interpretation	22	Interpretation consistent with results, balancing benefits and harms, and considering other relevant evidence	
**Other information**			
Registration	23	Registration number and name of trial registry	2
Protocol	24	Where the full trial protocol can be accessed, if available	1
Funding	25	Sources of funding and other support (such as supply of drugs), role of funders	18

*We strongly recommend reading this statement in conjunction with the CONSORT 2010 Explanation and Elaboration for important clarifications on all the items. If relevant, we also recommend reading CONSORT extensions for cluster randomised trials, non-inferiority and equivalence trials, non-pharmacological treatments, herbal interventions, and pragmatic trials. Additional extensions are forthcoming: for those and for up to date references relevant to this checklist, see www.consort-statement.org.

For peer review only

STANDARD PROTOCOL ITEMS: RECOMMENDATIONS FOR INTERVENTIONAL TRIALS

SPIRIT CHECKLIST STHLM3MRI Study, BMJ Open

SPIRIT 2013 Checklist: Recommended items to address in a clinical trial protocol and related documents*

Section/item	ROW NUMBER	Description
**Administrative information**		
Title	1	Descriptive title identifying the study design, population, interventions, and, if applicable, trial acronym
Trial registration	62	Trial identifier and registry name. If not yet registered, name of intended registry
	2b	All items from the World Health Organization Trial Registration Data Set
Protocol version	Full protocol	Date and version identifier
Funding	566	Sources and types of financial, material, and other support
Roles and responsibilities	7+559	Names, affiliations, and roles of protocol contributors
	20	Name and contact information for the trial sponsor
	Full protocol	Role of study sponsor and funders, if any, in study design; collection, management, analysis, and interpretation of data; writing of the report; and the decision to submit the report for publication, including whether they will have ultimate authority over any of these activities
	Full protocol	Composition, roles, and responsibilities of the coordinating centre, steering committee, endpoint adjudication committee, data management team, and other individuals or groups overseeing the trial, if applicable (see Item 21a for data monitoring committee)
**Introduction**		
Background and rationale	80, 267	Description of research question and justification for undertaking the trial, including summary of relevant studies (published and unpublished) examining benefits and harms for each intervention
	152	Explanation for choice of comparators

For peer review only - http://bmjopen.bmj.com/site/about/guidelines.xhtml

Objectives	225	Specific objectives or hypotheses
Trial design	275	Description of trial design including type of trial (eg, parallel group, crossover, factorial, single group), allocation ratio, and framework (eg, superiority, equivalence, noninferiority, exploratory)

Methods: Participants, interventions, and outcomes

Study setting	284	Description of study settings (eg, community clinic, academic hospital) and list of countries where data will be collected. Reference to where list of study sites can be obtained
Eligibility criteria	293	Inclusion and exclusion criteria for participants. If applicable, eligibility criteria for study centres and individuals who will perform the interventions (eg, surgeons, psychotherapists)
Interventions	319	Interventions for each group with sufficient detail to allow replication, including how and when they will be administered
	N/A	Criteria for discontinuing or modifying allocated interventions for a given trial participant (eg, drug dose change in response to harms, participant request, or improving/worsening disease)
	N/A	Strategies to improve adherence to intervention protocols, and any procedures for monitoring adherence (eg, drug tablet return, laboratory tests)
	N/A	Relevant concomitant care and interventions that are permitted or prohibited during the trial
Outcomes	424	Primary, secondary, and other outcomes, including the specific measurement variable (eg, systolic blood pressure), analysis metric (eg, change from baseline, final value, time to event), method of aggregation (eg, median, proportion), and time point for each outcome. Explanation of the clinical relevance of chosen efficacy and harm outcomes is strongly recommended
Participant timeline	450	Time schedule of enrolment, interventions (including any run-ins and washouts), assessments, and visits for participants. A schematic diagram is highly recommended (see Figure)
Sample size	453	Estimated number of participants needed to achieve study objectives and how it was determined, including clinical and statistical assumptions supporting any sample size calculations
Recruitment	480	Strategies for achieving adequate participant enrolment to reach target sample size

Methods: Assignment of interventions (for controlled trials)

Allocation:

For peer review only - http://bmjopen.bmj.com/site/about/guidelines.xhtml

Sequence generation	305	Method of generating the allocation sequence (eg, computer-generated random numbers), and list of any factors for stratification. To reduce predictability of a random sequence, details of any planned restriction (eg, blocking) should be provided in a separate document that is unavailable to those who enrol participants or assign interventions
Allocation concealment mechanism	305	Mechanism of implementing the allocation sequence (eg, central telephone; sequentially numbered, opaque, sealed envelopes), describing any steps to conceal the sequence until interventions are assigned
Implementation	315	Who will generate the allocation sequence, who will enrol participants, and who will assign participants to interventions
Blinding (masking)	N/A	Who will be blinded after assignment to interventions (eg, trial participants, care providers, outcome assessors, data analysts), and how
	N/A	If blinded, circumstances under which unblinding is permissible, and procedure for revealing a participant's allocated intervention during the trial
**Methods: Data collection, management, and analysis**		
Data collection methods	488	Plans for assessment and collection of outcome, baseline, and other trial data, including any related processes to promote data quality (eg, duplicate measurements, training of assessors) and a description of study instruments (eg, questionnaires, laboratory tests) along with their reliability and validity, if known. Reference to where data collection forms can be found, if not in the protocol
	N/A	Plans to promote participant retention and complete follow-up, including list of any outcome data to be collected for participants who discontinue or deviate from intervention protocols
Data management	498	Plans for data entry, coding, security, and storage, including any related processes to promote data quality (eg, double data entry; range checks for data values). Reference to where details of data management procedures can be found, if not in the protocol
Statistical methods	506 Full protocol	Statistical methods for analysing primary and secondary outcomes. Reference to where other details of the statistical analysis plan can be found, if not in the protocol
	Full protocol	Methods for any additional analyses (eg, subgroup and adjusted analyses)

	Full Protocol	Definition of analysis population relating to protocol non-adherence (eg, as randomised analysis), and any statistical methods to handle missing data (eg, multiple imputation)
**Methods: Monitoring**		
Data monitoring	508	Composition of data monitoring committee (DMC); summary of its role and reporting structure; statement of whether it is independent from the sponsor and competing interests; and reference to where further details about its charter can be found, if not in the protocol. Alternatively, an explanation of why a DMC is not needed
	508	Description of any interim analyses and stopping guidelines, including who will have access to these interim results and make the final decision to terminate the trial
Harms	441	Plans for collecting, assessing, reporting, and managing solicited and spontaneously reported adverse events and other unintended effects of trial interventions or trial conduct
Auditing	508	Frequency and procedures for auditing trial conduct, if any, and whether the process will be independent from investigators and the sponsor
**Ethics and dissemination**		
Research ethics approval	524	Plans for seeking research ethics committee/institutional review board (REC/IRB) approval
Protocol amendments	Full protocol	Plans for communicating important protocol modifications (eg, changes to eligibility criteria, outcomes, analyses) to relevant parties (eg, investigators, REC/IRBs, trial participants, trial registries, journals, regulators)
Consent or assent	Full protocol	Who will obtain informed consent or assent from potential trial participants or authorised surrogates, and how (see Item 32)
	Full protocol	Additional consent provisions for collection and use of participant data and biological specimens in ancillary studies, if applicable
Confidentiality	532	How personal information about potential and enrolled participants will be collected, shared, and maintained in order to protect confidentiality before, during, and after the trial
Declaration of interests	551	Financial and other competing interests for principal investigators for the overall trial and each study site
Access to data	544	Statement of who will have access to the final trial dataset, and disclosure of contractual agreements that limit such access for investigators

For peer review only - http://bmjopen.bmj.com/site/about/guidelines.xhtml

Ancillary and post-trial care	N/A	Provisions, if any, for ancillary and post-trial care, and for compensation to those who suffer harm from trial participation
Dissemination policy	537	Plans for investigators and sponsor to communicate trial results to participants, healthcare professionals, the public, and other relevant groups (eg, via publication, reporting in results databases, or other data sharing arrangements), including any publication restrictions
	537	Authorship eligibility guidelines and any intended use of professional writers
	537	Plans, if any, for granting public access to the full protocol, participant-level dataset, and statistical code
**Appendices**		
Informed consent materials	Appe nxid	Model consent form and other related documentation given to participants and authorised surrogates
Biological specimens	N/A	Plans for collection, laboratory evaluation, and storage of biological specimens for genetic or molecular analysis in the current trial and for future use in ancillary studies, if applicable

*It is strongly recommended that this checklist be read in conjunction with the SPIRIT 2013 Explanation & Elaboration for important clarification on the items. Amendments to the protocol should be tracked and dated. The SPIRIT checklist is copyrighted by the SPIRIT Group under the Creative Commons "Attribution-NonCommercial-NoDerivs 3.0 Unported" license.

For peer review only

BMJ Open

Does a novel diagnostic pathway including blood-based risk-prediction and MRI-targeted biopsies outperform prostate cancer screening using prostate-specific antigen and systematic prostate biopsies? – Protocol of the randomized study STHLM3MRI.

Journal:	*BMJ Open*
Manuscript ID	bmjopen-2018-027816.R2
Article Type:	Protocol
Date Submitted by the Author:	13-May-2019
Complete List of Authors:	Nordstrom, Tobias; Karolinska Inst, Dpt Medical Epidemiology and Biostatistics<br>Jäderling, Fredrik; Karolinska Institutet, Molecular Medicine and Surgery<br>Carlsson, Stefan; Karolinska Institutet<br>Aly, Markus; Karolinska Institutet,<br>Grönberg, H; Karolinska Institutet,<br>Eklund, Martin; Karolinska Institutet,
**Primary Subject Heading**:	Urology
Secondary Subject Heading:	Diagnostics
Keywords:	Prostate disease < UROLOGY, Magnetic resonance imaging < RADIOTHERAPY, Urological tumours < UROLOGY

SCHOLARONE™
Manuscripts

Title:

Does a novel diagnostic pathway including blood-based risk-prediction and MRI-targeted biopsies outperform prostate cancer screening using prostate-specific antigen and systematic prostate biopsies? – Protocol of the randomized study STHLM3MRI.

Authors:

Tobias Nordström[a,b], MD PhD

Fredrik Jäderling[e,f], MD PhD

Stefan Carlsson[d,f], MD PhD

Markus Aly[a,d,f], MD PhD

Henrik Grönberg[a], MD PhD, Professor

Martin Eklund[a], PhD

[a]Department of Medical Epidemiology and Biostatistics, Karolinska Institutet, Sweden

[b]Department of Clinical Sciences at Danderyd Hospital, Karolinska Institutet, Sweden

[d]Patient area Pelvic Cancer, Theme Cance Karolinska University Hospital Solna, Stockholm, Sweden

[e]Department of Diagnostic Radiology, Karolinska University Hospital, Stockholm,Sweden

[f]Department of Molecular Medicine and Surgery, Karolinska Institutet, Stockholm, Sweden

Correspondence to:

Tobias Nordström

Department of Medical Epidemiology and Biostatistics, Karolinska Institutet

S-171 77 Stockholm, Sweden

Email: tobias.nordstrom@ki.se

Phone: + 46 70 539 17 91

Keywords: Prostate cancer, Prostate neoplasm, biomarker, STHLM3, prostate biopsy, magnetic resonance imaging

For peer review only

1. Abstract

Introduction

Prostate cancer is a leading cause of cancer death among men in the Western world. Early detection of prostate cancer has been shown to decrease mortality, but has limitations with low specificity leading to unnecessary biopsies and over-diagnosis of low-risk cancers. The STHLM3 trial has paved way for improved specificity in early detection of prostate cancer using the blood-based STHLM3 test for identifying men at increased risk of harbouring significant prostate cancer. Targeted prostate biopsies based on MRI images have been shown non-inferior sensitivity to detect significant prostate cancer and decrease the number of biopsies and non-significant cancers among men referred for prostate biopsy in clinical practice.

The strategy of the STHLM3-MRI projects is to study an improved diagnostic pathway including an improved blood-based test for identification of men with increased risk of prostate cancer and use of MRI to select men for diagnostic workup with targeted prostate biopsies.

Methods

This study compares prostate cancer detection using PSA and systematic biopsies to the improved pathway for prostate cancer detection using the STHLM3 test and targeted biopsies in a screening context. The study will recruit 10,000 participants during June 1st 2018 to June 1st 2020 combining a paired and randomized design. Participants are grouped by PSA and Stockholm3 test level. Men with Stockholm3 ≥11% or PSA ≥3ng/ml are randomized to systematic or MRI-targeted biopsies. This protocol follows SPIRIT guidelines. Endpoints include the number of detected prostate cancers, number of performed biopsy procedures and number of performed MRIs. Additional aims include to assess the health economic consequences and development of automated image-analysis.

Ethics and dissemination

The study is approved by the regional ethical review board in Stockholm (2017-1280/31). Study findings will be published in peer-review journals. Findings will also be disseminated by conference/departmental presentations

For peer review only - http://bmjopen.bmj.com/site/about/guidelines.xhtml

and by media.

Registration details

ClinicalTrials.gov: NCT03377881

2. Strengths and limitations of this study

- This is the first randomized study to examine the role of improved blood-based risk stratification used in sequence with MRI and targeted prostate biopsies in a screening-by-invitation context.
- The study examines the performance of the Stockholm3 test used together with MRI/Fusion technique compared to traditional PSA screening and will provide important data also on the performance of the Stockholm3 test or MRI/Fusion when used as standalone strategies.
- The study is performed at three study sites and uses centralized radiology and pathology.
- The study is limited to a Swedish screening population, the use of the Stockholm3 test as blood-based risk prediction test and the technology used for MRI-targeted biopsies.

3. Trial identifier

ClinicalTrials.gov Identifier: NCT03377881

4. Introduction

4.1. Public health significance of prostate cancer

Prostate cancer is the most common cancer and the leading cause of cancer death among men in Sweden. In year 2011 over 10,000 men were diagnosed with prostate cancer and more than 2,500 died due to the disease, approximately 20% of these in the Stockholm region. Prostate cancer incidence rates in Sweden are now comparable to rates in countries that had an early introduction of PSA testing, while prostate cancer mortality rates are

For peer review only - http://bmjopen.bmj.com/site/about/guidelines.xhtml

higher than in most other countries[1]. With over 90,000 prevalent cases, the health burden and the costs on the health care system are substantial. While a number of risk factors have been proposed for prevention of prostate cancer, including diet and occupational exposures, the only factors conclusively shown to increase risk of the disease are age, ethnicity and family history. Given the high prevalence of the cancer and limited opportunities for primary prevention, improved detection would reduce both procedure-related harm to men and economical cost in the healthcare system.

4.2. Early detection and treatment of prostate cancer: benefits and harms

The PSA test was first used to monitor disease progression in prostate cancer patients. The PSA test was taken up as a *de facto* screening test for prostate cancer in many countries, leading to a rapid rise in prostate cancer incidence. The test characteristics for the PSA test in detecting prostate cancer are comparable to those for mammography for breast cancer screening, with a sensitivity of 72% and a specificity of 30-35% at a test threshold of 4 ng/ml[2]. However, a lower threshold of 3 ng/ml adopted in Sweden recently has led to increased sensitivity at the expense of reduced specificity. Recent analyses of PSA testing in the Stockholm area confirms these results showing that 46%, 68% and 77% of men 50-59, 60-69 and 70-79 years respectively have had at least one PSA test during a 9 years period[3].

Recent results from the large European Randomized Study of Screening for Prostate Cancer (ERSPC) including over 180,000 men provide increasing evidence that PSA screening has led to reduced mortality[4]. This report showed that PSA screening without digital rectal examination was associated with a 21% relative reduction in the death rate from prostate cancer at a median follow-up of 11 years, with an absolute reduction of about 7 prostate cancer deaths per 10,000 men screened. Estimations from the ERSPC trial (men aged 55-69) show that 1,048 men would need to be offered screening and an additional 37 would need to be managed to prevent one prostate-cancer death during a 10-year period, leading to a significant overtreatment of indolent disease. The effectiveness of PSA testing was more marked at the

For peer review only - http://bmjopen.bmj.com/site/about/guidelines.xhtml

Göteborg site of the ERSPC trial, with a risk reduction of 44% over 14 years in men aged 50-64[5]. This effect size is larger than that observed for mammographic screening for breast cancer and faecal occult blood testing for colorectal cancer.

However, using traditional systematic biopsies for diagnosis, approximately half of diagnosed cancers are low-risk tumours using the same main cut-off for biopsy as the ERSPC trial (PSA=3ng/ml) [6,7]. It has been shown that men with low-risk tumours treated without curative intent have the same survival as men in the background population[8], illustrating the large proportion of over-diagnosed cancers[9].

The STHLM3 study has shown one way to improve identification of men at increased risk of significant prostate cancer. Using the STHLM3 test, 32% of the prostate biopsies may be saved while not decreasing the sensitivity to high-grade disease (defined as Gleason Score ≥7) and simultaneously decreasing the number of low-grade tumours (Gleason Score ≤6) by 17%, thus decreasing overdiagnosis[7].

4.3. Traditional evaluation of men with increased risk of prostate cancer

Men at increased risk of prostate cancer - commonly estimated using PSA and palpatory findings - are traditionally assessed using systematic prostate biopsies. The procedure is performed under local anaesthesia using antibiotic prophylaxis and includes 10-12 cores taken from predefined areas of the peripheral zone of the gland as visualized by endorectal ultrasound. While the biopsies systematically covers the prostatic gland rather than targeting a specific lesion, and non-lethal tumours are common, the risk of over-diagnosis (i.e. detection of non-significant tumours) is high [9]. The risk of non-representative biopsy findings result in underestimation of tumour grade compared with subsequent prostatectomy in up to 40% of men undergoing surgery[10]. The risk of severe post-biopsy infection has increased to 1-2% with increasing frequency of antibiotic resistance, further illustrating the need both to increase precision and decrease the number of performed biopsies[11]. Since screening using PSA and systematic prostate biopsies

For peer review only

have been shown to decrease prostate cancer mortality, it is reasonable to use this strategy as comparator for novel diagnostic strategies[4-5].

4.4. Multi-parametric Magnetic Resonance Imaging (mpMRI) for detection of prostate cancer

Multi-parametric magnetic resonance imaging (mpMRI) incorporating anatomical and functional imaging has now been validated as a means of detecting and characterizing prostate tumours and can aid in risk stratification and treatment selection. The European Society of Urogenital Radiology (ESUR) in 2012 established the Prostate Imaging Reporting and Data System (PI-RADS) guidelines aimed at standardizing the acquisition, interpretation and reporting of prostate mpMRI. Consensus on an updated version (PI-RADS v2) have recently been published, outlining aspects of both interpretation and the technical execution[12-14]. Use of the revised PI-RADS provides moderately reproducible MR imaging scores for detection of clinically relevant disease[15]. Using MP-MRI to triage men might allow 27% of patients avoid a primary biopsy and diagnosis fewer clinically insignificant cancers. If subsequent TRUS-biopsies were directed by MP-MRI findings, up to 18% more cases of clinically significant cancer might be detected compared with the standard pathway of TRUS-biopsy for all[16].

In summary, PI-RADS recommends to use 3T or 1.5T machines, including T2- and T1-weighted sequences together with diffusion weighted images (DWI). Currently, the added value of dynamic contrast is not firmly established regarding tumour detection. At this time, there is no consensus among experts concerning the potential benefits of the use of endorectal coils for cancer detection. It has been suggested that the prevalence of suspicious lesions on MRI in men with clinical suspicion of prostate cancer is approximately 60% [17].

4.5. Targeted prostate biopsies guided by fusion technology

Targeted biopsies of the prostate consist of imaging (MRI) detecting significant tumours and a biopsy procedure where biopsies are targeted to the tumour using various devices for guidance[18]. While traditional endorectal ultrasound poorly identifies tumours, direction of biopsy needles can be

For peer review only - http://bmjopen.bmj.com/site/about/guidelines.xhtml

performed in various ways. Cognitive or soft fusion is based on skilled urologists/radiologists interpreting the MRI images and directing needles solely based on the ultrasound images. The disadvantages of cognitive fusion lie in the potential for human error when attempting to mentally fuse the MRI with TRUS while aiming for cancers that are often <1 cm in diameter and the inability to track the location of each biopsy site. Hard fusion enables proper fusion of MRI information on the ultrasound image, possibly increasing precision.

Despite methodological flaws, a number of studies have investigated the value of fusion biopsies, primarily using non-randomized designs and non-screening populations[19]. In 2018, Kasivisvanathan et al provided high quality evidence for men referred for prostate biopsy and showed that MRI/target biopsies are non-inferior for detection of significant cancer and decreases the number of in-significant cancers and number of biopsies as compared with systematic biopsies[20].

The proportion of men upgraded when comparing specimen from targeted biopsies and subsequent prostatectomy have been shown to be very low (<5%) when using targeted biopsies[21], increasing the proportion of men where treatment decisions are based on valid risk estimations.

4.6. Improving the diagnostic pathway for prostate cancer detection

The current diagnostic pathway for prostate cancer detection is characterized by several challenging hallmarks. First, testing with PSA is frequent also in men not benefitting from testing due to low PSA levels or high age[3]. Second, the currently used test for detection (PSA) lacks in specificity, resulting in frequent over-diagnosis[22,23]. Third, systematic biopsies shows high frequencies of benign tests, over-diagnosis, up-grading at prostatectomy, and risk of infectious complications[7,24]. Further, PSA testing increases with educational length and men with long education are more likely to have a prostate biopsy after an increased PSA value. These differences may contribute to the worse prostate cancer outcomes observed among men with lower socioeconomic status[25].

For peer review only - http://bmjopen.bmj.com/site/about/guidelines.xhtml

The STHLM3 test offers improved disease detection[7]. To further decrease over-detection, improve disease classification and spare men of test-related harm, prostate biopsy practices need to be improved. We hypothesize that an improved pathway for prostate cancer detection including a better blood-based screening test, improved selection to biopsy based on MRI findings and targeted biopsies guided by MRI/ultrasound fusion would dramatically decrease the number of biopsy procedures, overdiagnosis and improve treatment decisions.

5. Methods

5.1. Hypotheses

5.1.1. Primary hypotheses

The hypothesis below is posed for men in screening-by-invitation context:

A diagnostic pathway using the Stockholm3 test to select men for further workup using MRI followed by targeted biopsies and systematic biopsies (S3M-MR-TBx/SBx) has non-inferior sensitivity for detecting clinically significant cancer (ISUP grade group ≥ 2) and shows superior specificity (reduction in number of performed biopsy procedures and detected ISUP 1 tumours) compared to the diagnostic pathway using systematic biopsies in men with PSA ≥3 ng/ml (PSA-SBx).

5.1.2. Additional hypotheses

1. As compared with performing systematic biopsies for men with elevated risk of prostate cancer in prostate cancer screening, targeted prostate biopsies performed with MRI/Fusion technique with or without addition of systematic biopsies has non-inferior sensitivity for detecting clinically significant cancer (ISUP grade group ≥ 2) and reduces the number of performed biopsy procedures.
2. A diagnostic pathway using the Stockholm3 test to select men for further workup using MRI followed by ONLY targeted biopsies (S3M-

For peer review only

MR-TBx) has non-inferior sensitivity for detecting clinically significant cancer (ISUP grade group ≥ 2) and reduces the number of performed biopsy procedures compared to a diagnostic pathway using systematic biopsies in men with PSA ≥3 ng/ml (PSA-SBx).

3. Adding prostate volume as parameter in the diagnostic pathway with Stockholm3 test and MRI/Fusion biopsies improves model precision.
4. A diagnostic pathway with Stockholm3 followed by MRI and targeted biopsies has non-inferior sensitivity for detecting clinically significant cancer (ISUP grade group ≥ 2) and reduces the number of MRI examinations and performed biopsies compared to a diagnostic pathway using PSA ≥3 ng/ml followed by MRI and targeted biopsies.
5. SBx in the MRI arm has superior sensitive than SBx in the non-MRI arm (due to cognitive fusion).
6. Biopsy compliance is higher after biopsy is recommended based on MRI compared to recommended without MRI.
7. A diagnostic pathway using the Stockholm3 test to select men for further workup using MRI and targeted biopsies (S3M+TBx) shows better health economy (positive ICER) compared to a diagnostic pathway using systematic biopsies in men with PSA ≥3 ng/ml (PSA+SBx).

5.2. Aims

To compare a diagnostic pathway using the Stockholm3 test (S3M ≥ 11%) to select men for further workup using MRI (PI-RADS ≥ 3) and targeted biopsies (S3M+TBx) to a diagnostic pathway using systematic biopsies in men with PSA ≥3 ng/ml (PSA+SBx) with respect to number of diagnosed clinically significant cancer (ISUP grade group ≥ 2) and number of performed biopsies. Additional aims corresponding to hypotheses 2-8 above will be assessed.

5.3. Study design

For peer review only

STHLM3-MR Phase 2 is a study combining a paired and a randomized design (Figure 1). The study will follow the following outline: Participants will be invited by mail. All participants will undergo a blood-test, including PSA and the STHLM3 test. Men with an elevated PSA ≥3 ng/ml *or* PSA ≥1.5ng/ml and S3M>11% will be randomized to either traditional prostate biopsies or MR with targeted biopsies on MR lesions.

5.4. Participants, interventions and outcomes

5.4.1. Study setting

This is a screening-by-invitation study including one study administrative centre, two radiological sites and three urological sites where data will be collected.

Participating urological centres

Department of Urology, Capio St Görans Hospital: dr Henrik Grönberg

Uroclinic, Sophiahemmet, Stockholm; dr Olof Jansson

Odenplans läkarhus; dr Magnus Annerstedt

5.4.2. Eligibility criteria

Inclusion criteria

Men age 50-74 years without prior diagnosis of prostate cancer (ICD-9 C61).

Permanent postal address in Stockholm

Not a previous participant in the Stockholm3 study (2012-2014)

Exclusion criteria

Severe illnesses such as metastatic cancers, severe cardio-vascular disease or dementia

Contraindications for magnetic resonance imaging (MRI) e.g. pacemaker, magnetic cerebral clips, cochlear implants or severe claustrophobia.

Men with a previous prostate biopsy the preceding 60 days before invitation.

For peer review only - http://bmjopen.bmj.com/site/about/guidelines.xhtml

5.4.3. Randomization

Randomization is performed 2:3 between control arm and experimental arm. Randomization will be performed will be performed using stratification on disease risk [6 strata]. Disease risk is assessed using the Stockholm3 test. Test are discordant if PSA is negative and Stockholm3 positive or vice versa.

Four allocation lists [high/low risk vs discordant/concordant tests] have been created with the sequence [control arm, control arm, experimental arm, experimental arm, experimental arm]. Participants are first allocated to corresponding list, and then allocated to study arm according to the order in which they participate. The allocation sequence is blinded for the study investigators and handled by the study database administrator (A Björklund).

In order to enhance resource usage, men are allocated to the study sites according to local availability of biopsy procedure slots.

5.4.4 Interventions

Blood sampling

Participating men undergo blood-sampling with analysis of PSA and the Stockholm3 test at Karolinska University Laboratory.

For the main analysis, the Stockholm3 test include clinical data as answered when consenting participation (previous biopsy, age, finasteride medication, relatives with prostate cancer); single nucleotide polymorphisms and measurements of protein levels (MSMB, MIC1, PSA, fPSA, hK2)[7]. For secondary analyses, clinical information on DRE and prostate volume is included.

Definition of EXPERIMENTAL ARM

Men randomized to the experimental arm undergo MRI. If suspicious lesions are found, the participant undergoes targeted biopsies using Fusion technology *followed by systematic biopsies.*

For peer review only

For peer review only - http://bmjopen.bmj.com/site/about/guidelines.xhtml

Men without lesions are excepted from further intervention and receives notification on recommendation for follow-up. Technology and process are described below.

Men with a Stockholm3 risk ≥25% and no suspicious lesion on MRI will be recommended to undergo systematic biopsies.

Definition of CONTROL ARM

Men randomized to the control arm undergoes systematic biopsies as defined below.

5.4.5 Technology

Cut-offs for performing the STHLM3 test

The STHLM3 test will be performed for men with a PSA ≥ 1.5 ng/ml

Cut-offs for entering randomization

Participants with PSA ≥ 3.0 ng/ml or STHLM3-test ≥ 11% risk of Gleason Score ≥7 cancer will be randomized and offered to undergo either MR or systematic biopsies (See Process description).

MRI technology

Location and MRI equipment

Capio St Görans Hospital: General Electric, Architect, 3T
Globen Unilabs Healthcare: Siemens Magnetom Aera 1.5T

Patient preparations

Refraining from sexual activity with ejaculation 3 days prior to examination
Fasting patient 6 h
Minimal preparation enema prior to examination
Antispasmodic agent (Glucagon) just before the examination

MRI Protocol

A short (14 minutes) MRI protocol will be used. A detailed description is available. Briefly, the protocol includes: T2w images axial, sagittal, coronal;

For peer review only - http://bmjopen.bmj.com/site/about/guidelines.xhtml

Diffusion weighted imaging b0 and b1000 with ADC and a synthetic b1500 limited to the prostate location; Endorectal coil will not be used.

MRI Interpretation

MRI interpretation is centralized to Capio St Görans hospital and is performed according to PIRAD v2.0 for examinations without adequate perfusion studies. Dr Fredrik Jäderling is responsible for MRI interpretation. Dr Jäderling or one to two other, experienced radiologists at his department performs all MRI interpretations.

PI-RADS v2 ("Assessment without adequate dynamic contrast enhanced imaging") will be used, with a 1-5 grade scale of suspicious lesions (1= clinically significant cancer is highly unlikely to be present, 5= clinically significant cancer is highly likely to be present).

During the study period participating radiologist will have access to updated histology results of fusion biopsies to be able to adjust their MRI reading according to tumour detection rates for different PIRAD scores as defined above.

Fusion biopsy technology

Brand/models

BK Medical (BK Ultrasound ; www.bkultrasound.com/bk-medical/fusion)

The BK Medical fusion system is the only fusion device compatible with BK Medicals ultrasound devices, used by the urology departments participating in the study. The system represents a second-generation ultrasound system with integrated MRI Fusion. MRI data is imported through HIPAA-compliant PACS connection with the local radiology department.

Definition of targeted biopsies

Using MRI data with pre-marked borders of the prostate and tumour, fusion of MRI images and ultrasound images are performed bedside. Using local anaesthetics and antibiotic prophylaxis, lesions are taken according to the

For peer review only - http://bmjopen.bmj.com/site/about/guidelines.xhtml

schedule below. Targeted biopsies are always combined with systematic biopsies.

Biopsy procedure for targeted biopsies

PI-RADS≥3: 3-4 targeted biopsies on marked lesions + systematic biopsies.

Large diffuse lesions or poor image quality: Systematic biopsies including lesion.

No PI-RADS≥3, diffuse lesions and at least acceptable image quality: No biopsies are performed.

In larger lesions in PI-RADS category 3 and 5, areas within the lesion with the lowest ADC value ("Target-within-target") will be targeted with the first biopsy taken from the lesion, to evaluate the additional value regarding tumour staging.

Definition of systematic biopsies

10-12 systematic biopsies are taken from the peripheral zone as previously described in STLHLM3 and the National Guidelines. Extra biopsies are allowed from additional sites visible on ultrasound or according to palpatory findings. In summary, systematic biopsies are performed in the peripheral zone as 4 lateral and para-median biopsies on the left and right side, in the base and mid part of the gland. In the apical third of the gland one lateral left and right biopsy is performed.

Pathology

Pathology is centralized to Unilabs/Capio St Görans hospital. Dr Axel Glaessgen is responsible for the integrity of analyses of pathological specimen. 2-3 uro-pathologists at dr Glaessgens department assesses all pathological specimen with intermittent cross-validation between them. Pathology preparation and reporting follow ISUP 2014 guidelines.

The pathology preparation is done by Unilabs as part of the normal clinical routine. Biopsy specimens are analysed according to local practice.

For peer review only

Localisation of biopsies in the prostate are described using Swedish National Guideline nomenclature (A1-4; B1-4; C1-4; anterior/posterior). Gleason Score, mm cancer and % Gleason 4 is reported on each needle specimen.

Pathologist notes results in the usual way in the laboratory system. The result of the pathological analysis is submitted in accordance with existing clinical routines to the referring urologist. A copy of the result is delivered to the study administration.

5.4.4. Outcomes

There are three co-primary endpoints in this trial: (i) Number of diagnosed ISUP grade group ≥ 2 cancers; (ii) Number of diagnosed ISUP grade group 1 cancers; (iii) Number of performed biopsies.

5.4.5. Follow-up

Main study outcomes are assessed after prostate biopsy procedures. Additional participant data will be secured in the following circumstances:

No suspicious lesion on MRI

Men in the experimental arm without suspicious lesions on MRI will be informed and recommended follow-up by the responsible, local urologist. After additional ethical application, the co-investigators may initiate retrospective follow-up of these participants.

Men with diagnosed prostate cancer

Participants with prostate cancer diagnosed on biopsy within the study will be followed up after the biopsy to secure data on the following: Treatment modality (Active Surveillance, Surgery, Radiation); Treatment lead-time and site; Pathological report after surgery (positive margins, T-stage, etc). Data will be assessed through medical records intermittently.

5.5. Serious adverse events

For peer review only

Study nurse will monitor serious adverse events after the prostate biopsy procedures. To ensure this, the study nurse will follow this check medical journals for hospitalization within 1 week after the biopsy procedure in the journal systems Take Care and Cosmic (covering all hospitals in the Stockholm region). This will be initiated as individual biopsy results are registered at the study administration. Results will be provided to the Data Safety and Monitoring Board.

5.6. Participant timeline

Figure 2 illustrates the approximate timeline for participating men in STHLM3MRI Main Study.

5.7. Sample size

STHLM3-MR/Fusion Phase 2 will invite 25,000 men and aim to include 10,000 participants. We anticipate to perform 1,039 biopsy procedures altogether. Inclusion will continue until complete data on 415 men in the control arm (SBx) and 623 men in the experimental arm (MR-TBx-SBx).

Basic data and assumptions used in the sample size calculations

We used data from the STHLM3 trial for sample size calculations [7]. In this data, 18% of men with PSA ≥ 3 had a clinically significant prostate cancer when biopsied with SBx. We further noted that rTPR=1.45 for clinically significant prostate cancer comparing MRI+TBx with SBx based on the results from the PRECISION randomized trial [20]. However, we will for sample size calculations use rTPR=1.25 for MRI+TBx vs. SBx as a more conservative estimate. We set the non-inferiority delta to 4 percentage points for demonstrating noninferiority with respect to sensitivity of clinically significant prostate cancer. We set the alpha to 5%.

Primary contrast

Simulating 1000 trials (by bootstrapping from the STHLM3 data) under the assumptions outlined in the preceding section 303 men need to biopsied in the SBx arm based on PSA ≥ 3 to have 80% power to demonstrate non-

For peer review only

inferior sensitivity of S3M+MRI+TBx compared with PSA+SBx. This means that at least **415** men need to be biopsied in the SBx arm (since some men are not randomized based on PSA ≥ 3 but on S3M ≥ 11%) and, consequently, **623** to the MRI arm (because of the 2:3 randomization). Total number of men undergoing workup according to protocol (SBx in the no MRI arm and MRI and TBx if Pi-RADS ≥ 3 in the MRI arm) is thus 1038. Assuming 20% dropout, 1300 men need to be randomized. These numbers give 80% power to detect a modest 17% reduction in biopsies between the two strategies.

5.8. Recruitment and Process Description

The STHLM3-MR Phase 2 will use existing solutions developed and optimized in the previous studies STHLM3 and STHLM3-MR Phase 1 where all major components of the process have been tested. First, participants will follow the *paired design study process* where inclusion, blood-test and delivery of recommendation letter is performed. Men with increased risk of high-grade prostate cancer then enter the *randomized study process*, where extended work-up including biopsies are performed.

5.9. Data Collection, management, analysis

5.9.1. Data collection

Primary data sources are

i. clinical variables collected from laboratory referral
ii. biopsy referrals and reports
iii. pathology reports
iv. MRI reports
v. blood concentrations of kallikreins, MSMB, MIC1, SNPs

Collection of i. – iv. is performed by study nurses (C Cavalli-Björkman) on a weekly basis from participating urology sites, participating radiologists. For v., this is digitally transferred from Karolinska University Laboratory.

5.9.2. Data management

Data is collected, entered, coded and stored at Department of Medical Epidemiology and Biostatistics, Karolinska Institutet. Data is entered by Study Nurse using predefined database sheets developed in STHLM3MRI Phase 1.

For peer review only - http://bmjopen.bmj.com/site/about/guidelines.xhtml

This is blinded from study co-investigators and data is stored at the department under supervision by the study database administrator (SDA, Astrid Björklund). Any extraction of study data is performed by the SDA after approval of PI Tobias Nordström.

5.9.3. Data analysis

Analysis of data is described in the Statistical Analysis Plan (SAP).

5.9.4. Auditing and Monitoring

A Data Safety and Monitoring Board (DSMB) is assembled and consist of dr Hans Garmo (Statistician), prof Ola Bratt (Urology) and prof Holmberg (Urology/Study Design). The DSMB audits protocol and process descriptions and one interim data extraction performed by the study database administrator after 10% (100 men) have completed the control or experimental arms. The co-investigators are blinded to the interim data and analysis results. The work of the DSMB is regulated in the DSMB Charter.

5.10. Patient and Public Involvement

The research question and outcome measures were designed to improve prostate cancer diagnostics. This includes optimizing prostate biopsies and decreasing over-detection, both associated with morbidity. Patient organisations were informed on the results from the STHLM3MRI Phase 1 study. Patients were not involved in recruitment of the study. Results will be disseminated to participants through common and scientific channels.

6. Ethics and dissemination

6.1. Research ethics approval

The study has approval from the regional ethical review board Regional Ethical Review Board in Stockholm (2017-1280/31).

6.2. Consent

For peer review only

Participant consent is secured when the participant is included to the study at www.kliniskastudier.se. This includes secure identification using Mobilt BankID. Additional approval on use of biological specimen data is collected on the biopsy referral.

6.3. Confidentiality

Study data is collected and stored at Department of Medical Epidemiology and Biostatistics, Karolinska Institutet using secure Oracle servers. All data extractions are made by database administrator and are anonymized (personal id number is removed) before dissemination to researchers.

6.4. Dissemination

Analyses results on the posed aims will be submitted for peer-reviewed publication and submitted for presentation at scientific congress. Communication of the results will be made to patient organizations (Prostatacancerförbundet) and non-scientific channels. No use of professional writers is planned.

The study protocol is made publicly available through clinicaltrials.gov.

6.5. Data Sharing Statement

Anonymized, individual participant data that underlie the results reported in this article, after deidentification (text, tables, figures and appendices) will be available for data sharing. Proposals may be submitted up to 36 months following article publication. Data will be shared with investigators whose proposed use of the data has been approved by an independent review committee identified for this purpose.

7. Declarations of interest

Henrik Grönberg has five prostate cancer diagnostic related patents pending, has patent applications licensed to Thermo Fisher Scientific, and might receive royalties from sales related to these patents. Martin Eklund is named on four of these five patent applications. Karolinska Institutet

For peer review only - http://bmjopen.bmj.com/site/about/guidelines.xhtml

collaborates with Thermo Fisher Scientific in developing the technology for the Stockholm3 test.

8. Contributions

TN was the Principal investigator. TN, HG, ME, SC and MA designed the study. ME and TN interpreted preliminary data. FJ designed MRI protocols and collected data.

We thank participants, study organizers, participating researchers and clinicians, and patient advisers for their contributions to the STHLM3MRI project.

9. Funding statement

Funding was provided by the Swedish Cancer Society, (Cancerfonden), the Swedish Research Council (Vetenskapsrådet), Swedish Research Council for Health Working Life and Welfare (FORTE), The Strategic Research Programme on Cancer (StratCan), Karolinska Institutet, Swedish e-Science Research Center (SeRC) and Stockholm City Council (SLL). The STHLM3 study is a part of the Linnaeus Center CRISP ‘‘Predication and prevention of breast and prostate cancer’’ funded by the Swedish Research Council.

10. Figure legends

Figure 1: Study design overview STHLM3MRI Main Study

Figure 2: Timeline overview for study participants in STHLM3MRI Main Study

11. References

1 Ferlay J, Soerjomataram I, Dikshit R, *et al.* Cancer incidence and mortality worldwide: Sources, methods and major patterns in GLOBOCAN 2012. *Int J Cancer* 2014;:n/a–n/a. doi:10.1002/ijc.29210

2 Mistry K, Cable G. Meta-analysis of prostate-specific antigen and digital rectal examination as screening tests for prostate carcinoma. *J Am Board Fam Pract* 2003;**16**:95–101.

For peer review only

3 Nordström T, Aly M, Clements MS, *et al.* Prostate-specific antigen (PSA) testing is prevalent and increasing in Stockholm County, Sweden, Despite no recommendations for PSA screening: results from a population-based study, 2003-2011. *Eur Urol* 2013;**63**:419–25. doi:10.1016/j.eururo.2012.10.001

4 Schröder FH, Hugosson J, Roobol MJ, *et al.* Prostate-cancer mortality at 11 years of follow-up. *N Engl J Med* 2012;**366**:981–90. doi:10.1056/NEJMoa1113135

5 Hugosson J, Carlsson S, Aus G, *et al.* Mortality results from the Göteborg randomised population-based prostate-cancer screening trial. *Lancet Oncol* 2010;**11**:725–32. doi:10.1016/S1470-2045(10)70146-7

6 Nationell kvalitetsrapport för diagnosår 2012. Regionala Cancercentrum i Samverkan 2013. http://npcr.se/wp-content/uploads/2013/04/20131121-NPCR-Rapport-2012.pdf

7 Grönberg H, Adolfsson J, Aly M, *et al.* Prostate cancer screening in men aged 50-69 years (STHLM3): a prospective population-based diagnostic study. *Lancet Oncol* 2015;**16**:1667–76.

8 Rider JR, Sandin F, Andrén O, *et al.* Long-term outcomes among noncuratively treated men according to prostate cancer risk category in a nationwide, population-based study. *Eur Urol* 2013;**63**:88–96. doi:10.1016/j.eururo.2012.08.001

9 Loeb S, Bjurlin MA, Nicholson J, *et al.* Overdiagnosis and overtreatment of prostate cancer. *Eur Urol* 2014;**65**:1046–55. doi:10.1016/j.eururo.2013.12.062

10 Soares R, Di Benedetto A, Dovey Z, *et al.* Minimum 5-year follow-up of 1138 consecutive laparoscopic radical prostatectomies. *BJU Int* 2015;**115**:546–53. doi:10.1111/bju.12887

11 Aly M, Dyrdak R, Nordström T, *et al.* Rapid increase in multidrug-resistant enteric bacilli blood stream infection after prostate biopsy-A 10-year population-based cohort study. *Prostate* 2015;**75**:947–56. doi:10.1002/pros.22979

12 Barrett T, Turkbey B, Choyke PL. PI-RADS version 2: what you need to know. *Clin Radiol* 2015;**70**:1165–76. doi:10.1016/j.crad.2015.06.093

13 Barentsz JO, Weinreb JC, Verma S, *et al.* Synopsis of the PI-RADS v2 Guidelines for Multiparametric Prostate Magnetic Resonance Imaging and Recommendations for Use. *Eur Urol* 2015;**69**:41–9. doi:10.1016/j.eururo.2015.08.038

14 Weinreb JC, Barentsz JO, Choyke PL, *et al.* PI-RADS Prostate Imaging - Reporting and Data System: 2015, Version 2. *Eur Urol* 2015;**69**:16–40. doi:10.1016/j.eururo.2015.08.052

For peer review only - http://bmjopen.bmj.com/site/about/guidelines.xhtml

15 Muller BG, Shih JH, Sankineni S, *et al.* Prostate Cancer: Interobserver Agreement and Accuracy with the Revised Prostate Imaging Reporting and Data System at Multiparametric MR Imaging. *Radiology* 2015;**277**:741–50. doi:10.1148/radiol.2015142818

16 Ahmed HU, El-Shater Bosaily A, Brown LC, *et al.* Diagnostic accuracy of multi-parametric MRI and TRUS biopsy in prostate cancer (PROMIS): a paired validating confirmatory study. *Lancet* Published Online First: 19 January 2017. doi:10.1016/S0140-6736(16)32401-1

17 Moore CM, Robertson NL, Arsanious N, *et al.* Image-guided prostate biopsy using magnetic resonance imaging-derived targets: a systematic review. *Eur Urol* 2013;**63**:125–40. doi:10.1016/j.eururo.2012.06.004

18 Sonn GA, Margolis DJ, Marks LS. Target detection: magnetic resonance imaging-ultrasound fusion-guided prostate biopsy. *Urol Oncol* 2014;**32**:903–11. doi:10.1016/j.urolonc.2013.08.006

19 Schoots IG, Roobol MJ, Nieboer D, *et al.* Magnetic Resonance Imaging-targeted Biopsy May Enhance the Diagnostic Accuracy of Significant Prostate Cancer Detection Compared to Standard Transrectal Ultrasound-guided Biopsy: A Systematic Review and Meta-analysis. *Eur Urol* 2014;**68**:438–50. doi:10.1016/j.eururo.2014.11.037

20 Kasivisvanathan V, Rannikko AS, Borghi M, *et al.* MRI-Targeted or Standard Biopsy for Prostate-Cancer Diagnosis. *N Engl J Med* 2018;:NEJMoa1801993. doi:10.1056/NEJMoa1801993

21 Baco E, Ukimura O, Rud E, *et al.* Magnetic resonance imaging-transectal ultrasound image-fusion biopsies accurately characterize the index tumor: correlation with step-sectioned radical prostatectomy specimens in 135 patients. *Eur Urol* 2015;**67**:787–94. doi:10.1016/j.eururo.2014.08.077

22 Arnsrud Godtman R, Holmberg E, Lilja H, *et al.* Opportunistic Testing Versus Organized Prostate-specific Antigen Screening: Outcome After 18 Years in the Göteborg Randomized Population-based Prostate Cancer Screening Trial. *Eur Urol* 2014;**68**:354–60. doi:10.1016/j.eururo.2014.12.006

23 Thompson IM, Pauler DK, Goodman PJ, *et al.* Prevalence of prostate cancer among men with a prostate-specific antigen level. *N Engl J Med* 2004;**350**:2239–46. doi:10.1056/NEJMoa031918

24 Loeb S, Vellekoop A, Ahmed HU, *et al.* Systematic review of complications of prostate biopsy. *Eur Urol* 2013;**64**:876–92. doi:10.1016/j.eururo.2013.05.049

25 Nordström T, Bratt O, Örtegren J, *et al.* A population-based study on the association between educational length, prostate-specific antigen testing and use of prostate biopsies. *Scand J Urol* 2016;**50**:104–9. doi:10.3109/21681805.2015.1113200

For peer review only

For peer review only

For peer review only - http://bmjopen.bmj.com/site/about/guidelines.xhtml

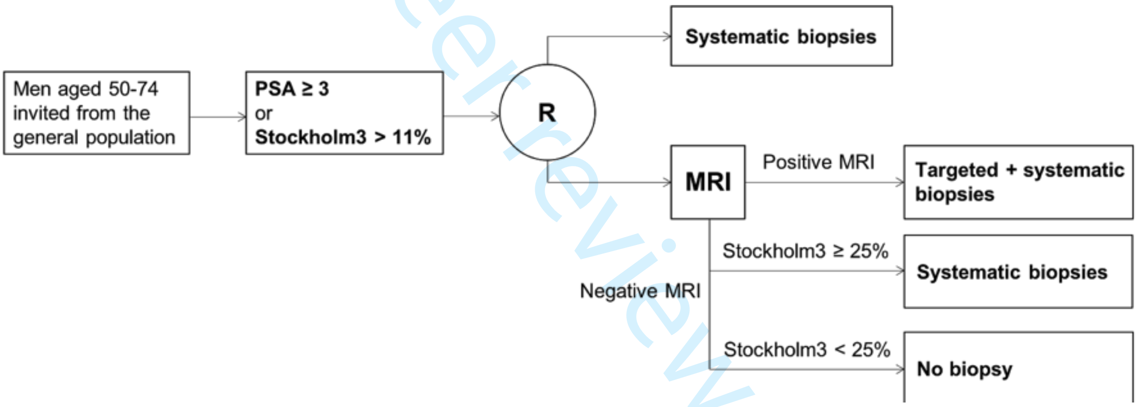
Men aged 50-74 invited from the general population
PSA ≥ 3 or Stockholm3 > 11%
R
Systematic biopsies
MRI
Positive MRI
Targeted + systematic biopsies
Negative MRI
Stockholm3 ≥ 25%
Systematic biopsies
Stockholm3 < 25%
No biopsy

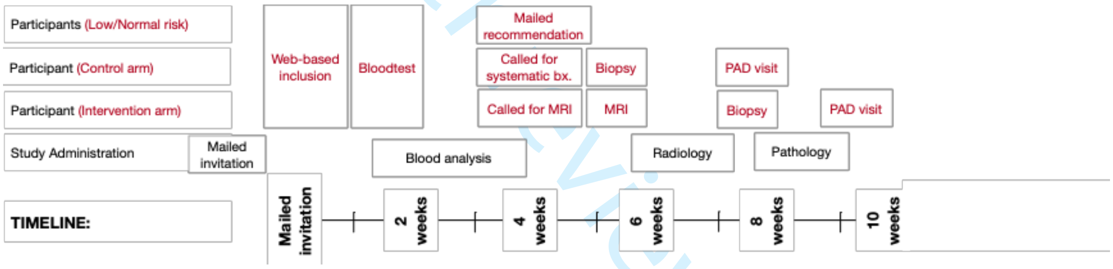
Participants (Low/Normal risk)
Participant (Control arm)
Participant (Intervention arm)
Study Administration
Mailed invitation
TIMELINE:
Web-based inclusion
Bloodtest
Mailed recommendation
Called for systematic bx.
Called for MRI
Biopsy
MRI
PAD visit
Biopsy
PAD visit
Blood analysis
Radiology
Pathology
Mailed invitation
2 weeks
4 weeks
6 weeks
8 weeks
10 weeks

CONSORT 2010 checklist of information to include when reporting a randomised trial*

Section/Topic	Item No	Checklist item	Reported on page No
**Title and abstract**			
	1a	Identification as a randomised trial in the title	1
	1b	Structured summary of trial design, methods, results, and conclusions (for specific guidance see CONSORT for abstracts)	1
**Introduction**			
Background and objectives	2a	Scientific background and explanation of rationale	4-7
	2b	Specific objectives or hypotheses	8-9
**Methods**			
Trial design	3a	Description of trial design (such as parallel, factorial) including allocation ratio	9
	3b	Important changes to methods after trial commencement (such as eligibility criteria), with reasons	10
Participants	4a	Eligibility criteria for participants	10
	4b	Settings and locations where the data were collected	9
Interventions	5	The interventions for each group with sufficient details to allow replication, including how and when they were actually administered	10-13
Outcomes	6a	Completely defined pre-specified primary and secondary outcome measures, including how and when they were assessed	14
	6b	Any changes to trial outcomes after the trial commenced, with reasons	
Sample size	7a	How sample size was determined	15
	7b	When applicable, explanation of any interim analyses and stopping guidelines	16
Randomisation:			
Sequence generation	8a	Method used to generate the random allocation sequence	-
	8b	Type of randomisation; details of any restriction (such as blocking and block size)	10
Allocation concealment mechanism	9	Mechanism used to implement the random allocation sequence (such as sequentially numbered containers), describing any steps taken to conceal the sequence until interventions were assigned	10
Implementation	10	Who generated the random allocation sequence, who enrolled participants, and who assigned participants to interventions	
Blinding	11a	If done, who was blinded after assignment to interventions (for example, participants, care providers, those	-

For peer review only - http://bmjopen.bmj.com/site/about/guidelines.xhtml

		assessing outcomes) and how	
	11b	If relevant, description of the similarity of interventions	
Statistical methods	12a	Statistical methods used to compare groups for primary and secondary outcomes	Separate doc
	12b	Methods for additional analyses, such as subgroup analyses and adjusted analyses	Separate doc
**Results**			
Participant flow (a diagram is strongly recommended)	13a	For each group, the numbers of participants who were randomly assigned, received intended treatment, and were analysed for the primary outcome	
	13b	For each group, losses and exclusions after randomisation, together with reasons	
Recruitment	14a	Dates defining the periods of recruitment and follow-up	
	14b	Why the trial ended or was stopped	
Baseline data	15	A table showing baseline demographic and clinical characteristics for each group	
Numbers analysed	16	For each group, number of participants (denominator) included in each analysis and whether the analysis was by original assigned groups	
Outcomes and estimation	17a	For each primary and secondary outcome, results for each group, and the estimated effect size and its precision (such as 95% confidence interval)	
	17b	For binary outcomes, presentation of both absolute and relative effect sizes is recommended	
Ancillary analyses	18	Results of any other analyses performed, including subgroup analyses and adjusted analyses, distinguishing pre-specified from exploratory	
Harms	19	All important harms or unintended effects in each group (for specific guidance see CONSORT for harms)	
**Discussion**			
Limitations	20	Trial limitations, addressing sources of potential bias, imprecision, and, if relevant, multiplicity of analyses	
Generalisability	21	Generalisability (external validity, applicability) of the trial findings	
Interpretation	22	Interpretation consistent with results, balancing benefits and harms, and considering other relevant evidence	
**Other information**			
Registration	23	Registration number and name of trial registry	2
Protocol	24	Where the full trial protocol can be accessed, if available	1
Funding	25	Sources of funding and other support (such as supply of drugs), role of funders	18

*We strongly recommend reading this statement in conjunction with the CONSORT 2010 Explanation and Elaboration for important clarifications on all the items. If relevant, we also recommend reading CONSORT extensions for cluster randomised trials, non-inferiority and equivalence trials, non-pharmacological treatments, herbal interventions, and pragmatic trials. Additional extensions are forthcoming: for those and for up to date references relevant to this checklist, see www.consort-statement.org.

For peer review only - http://bmjopen.bmj.com/site/about/guidelines.xhtml

SPIRIT
STANDARD PROTOCOL ITEMS: RECOMMENDATIONS FOR INTERVENTIONAL TRIALS

SPIRIT CHECKLIST STHLM3MRI Study, BMJ Open

SPIRIT 2013 Checklist: Recommended items to address in a clinical trial protocol and related documents*

Section/item	ROW NUMBER	Description
**Administrative information**		
Title	1	Descriptive title identifying the study design, population, interventions, and, if applicable, trial acronym
Trial registration	62	Trial identifier and registry name. If not yet registered, name of intended registry
	2b	All items from the World Health Organization Trial Registration Data Set
Protocol version	Full protocol	Date and version identifier
Funding	566	Sources and types of financial, material, and other support
Roles and responsibilities	7+559	Names, affiliations, and roles of protocol contributors
	20	Name and contact information for the trial sponsor
	Full protocol	Role of study sponsor and funders, if any, in study design; collection, management, analysis, and interpretation of data; writing of the report; and the decision to submit the report for publication, including whether they will have ultimate authority over any of these activities
	Full protocol	Composition, roles, and responsibilities of the coordinating centre, steering committee, endpoint adjudication committee, data management team, and other individuals or groups overseeing the trial, if applicable (see Item 21a for data monitoring committee)
**Introduction**		
Background and rationale	80, 267	Description of research question and justification for undertaking the trial, including summary of relevant studies (published and unpublished) examining benefits and harms for each intervention
	152	Explanation for choice of comparators

For peer review only

Objectives	225	Specific objectives or hypotheses
Trial design	275	Description of trial design including type of trial (eg, parallel group, crossover, factorial, single group), allocation ratio, and framework (eg, superiority, equivalence, noninferiority, exploratory)

Methods: Participants, interventions, and outcomes

Study setting	284	Description of study settings (eg, community clinic, academic hospital) and list of countries where data will be collected. Reference to where list of study sites can be obtained
Eligibility criteria	293	Inclusion and exclusion criteria for participants. If applicable, eligibility criteria for study centres and individuals who will perform the interventions (eg, surgeons, psychotherapists)
Interventions	319	Interventions for each group with sufficient detail to allow replication, including how and when they will be administered
	N/A	Criteria for discontinuing or modifying allocated interventions for a given trial participant (eg, drug dose change in response to harms, participant request, or improving/worsening disease)
	N/A	Strategies to improve adherence to intervention protocols, and any procedures for monitoring adherence (eg, drug tablet return, laboratory tests)
	N/A	Relevant concomitant care and interventions that are permitted or prohibited during the trial
Outcomes	424	Primary, secondary, and other outcomes, including the specific measurement variable (eg, systolic blood pressure), analysis metric (eg, change from baseline, final value, time to event), method of aggregation (eg, median, proportion), and time point for each outcome. Explanation of the clinical relevance of chosen efficacy and harm outcomes is strongly recommended
Participant timeline	450	Time schedule of enrolment, interventions (including any run-ins and washouts), assessments, and visits for participants. A schematic diagram is highly recommended (see Figure)
Sample size	453	Estimated number of participants needed to achieve study objectives and how it was determined, including clinical and statistical assumptions supporting any sample size calculations
Recruitment	480	Strategies for achieving adequate participant enrolment to reach target sample size

Methods: Assignment of interventions (for controlled trials)

Allocation:

For peer review only - http://bmjopen.bmj.com/site/about/guidelines.xhtml

Sequence generation	305	Method of generating the allocation sequence (eg, computer-generated random numbers), and list of any factors for stratification. To reduce predictability of a random sequence, details of any planned restriction (eg, blocking) should be provided in a separate document that is unavailable to those who enrol participants or assign interventions
Allocation concealment mechanism	305	Mechanism of implementing the allocation sequence (eg, central telephone; sequentially numbered, opaque, sealed envelopes), describing any steps to conceal the sequence until interventions are assigned
Implementation	315	Who will generate the allocation sequence, who will enrol participants, and who will assign participants to interventions
Blinding (masking)	N/A	Who will be blinded after assignment to interventions (eg, trial participants, care providers, outcome assessors, data analysts), and how
	N/A	If blinded, circumstances under which unblinding is permissible, and procedure for revealing a participant's allocated intervention during the trial
**Methods: Data collection, management, and analysis**		
Data collection methods	488	Plans for assessment and collection of outcome, baseline, and other trial data, including any related processes to promote data quality (eg, duplicate measurements, training of assessors) and a description of study instruments (eg, questionnaires, laboratory tests) along with their reliability and validity, if known. Reference to where data collection forms can be found, if not in the protocol
	N/A	Plans to promote participant retention and complete follow-up, including list of any outcome data to be collected for participants who discontinue or deviate from intervention protocols
Data management	498	Plans for data entry, coding, security, and storage, including any related processes to promote data quality (eg, double data entry; range checks for data values). Reference to where details of data management procedures can be found, if not in the protocol
Statistical methods	506 Full protocol	Statistical methods for analysing primary and secondary outcomes. Reference to where other details of the statistical analysis plan can be found, if not in the protocol
	Full protocol	Methods for any additional analyses (eg, subgroup and adjusted analyses)

	Full Protocol	Definition of analysis population relating to protocol non-adherence (eg, as randomised analysis), and any statistical methods to handle missing data (eg, multiple imputation)
**Methods: Monitoring**		
Data monitoring	508	Composition of data monitoring committee (DMC); summary of its role and reporting structure; statement of whether it is independent from the sponsor and competing interests; and reference to where further details about its charter can be found, if not in the protocol. Alternatively, an explanation of why a DMC is not needed
	508	Description of any interim analyses and stopping guidelines, including who will have access to these interim results and make the final decision to terminate the trial
Harms	441	Plans for collecting, assessing, reporting, and managing solicited and spontaneously reported adverse events and other unintended effects of trial interventions or trial conduct
Auditing	508	Frequency and procedures for auditing trial conduct, if any, and whether the process will be independent from investigators and the sponsor
**Ethics and dissemination**		
Research ethics approval	524	Plans for seeking research ethics committee/institutional review board (REC/IRB) approval
Protocol amendments	Full protocol	Plans for communicating important protocol modifications (eg, changes to eligibility criteria, outcomes, analyses) to relevant parties (eg, investigators, REC/IRBs, trial participants, trial registries, journals, regulators)
Consent or assent	Full protocol	Who will obtain informed consent or assent from potential trial participants or authorised surrogates, and how (see Item 32)
	Full protocol	Additional consent provisions for collection and use of participant data and biological specimens in ancillary studies, if applicable
Confidentiality	532	How personal information about potential and enrolled participants will be collected, shared, and maintained in order to protect confidentiality before, during, and after the trial
Declaration of interests	551	Financial and other competing interests for principal investigators for the overall trial and each study site
Access to data	544	Statement of who will have access to the final trial dataset, and disclosure of contractual agreements that limit such access for investigators

For peer review only

Ancillary and post-trial care	N/A	Provisions, if any, for ancillary and post-trial care, and for compensation to those who suffer harm from trial participation
Dissemination policy	537	Plans for investigators and sponsor to communicate trial results to participants, healthcare professionals, the public, and other relevant groups (eg, via publication, reporting in results databases, or other data sharing arrangements), including any publication restrictions
	537	Authorship eligibility guidelines and any intended use of professional writers
	537	Plans, if any, for granting public access to the full protocol, participant-level dataset, and statistical code
**Appendices**		
Informed consent materials	Appe nxid	Model consent form and other related documentation given to participants and authorised surrogates
Biological specimens	N/A	Plans for collection, laboratory evaluation, and storage of biological specimens for genetic or molecular analysis in the current trial and for future use in ancillary studies, if applicable

*It is strongly recommended that this checklist be read in conjunction with the SPIRIT 2013 Explanation & Elaboration for important clarification on the items. Amendments to the protocol should be tracked and dated. The SPIRIT checklist is copyrighted by the SPIRIT Group under the Creative Commons "Attribution-NonCommercial-NoDerivs 3.0 Unported" license.

For peer review only
